# Targeting substrate-site in Jak2 kinase prevents emergence of genetic resistance

**DOI:** 10.1038/srep14538

**Published:** 2015-09-30

**Authors:** Meenu Kesarwani, Erika Huber, Zachary Kincaid, Chris R. Evelyn, Jacek Biesiada, Mark Rance, Mahendra B. Thapa, Neil P. Shah, Jarek Meller, Yi Zheng, Mohammad Azam

**Affiliations:** 1 Cincinnati Children’s Hospital Medical Center, Cancer Blood Disease Institute, Divisions of Experimental Hematology and Cancer Pathology, Cincinnati, Ohio, 45229 USA; 2 Department of Molecular Genetics, Biochemistry and Microbiology University of Cincinnati College of Medicine, University of Cincinnati, Ohio 45229 USA; 3 Division of Hematology-Oncology UCSF School of Medicine, San Francisco, California, 94143 USA

## Abstract

Emergence of genetic resistance against kinase inhibitors poses a great challenge for durable therapeutic response. Here, we report a novel mechanism of JAK2 kinase inhibition by fedratinib (TG101348) that prevents emergence of genetic resistance. Using *in vitro* drug screening, we identified 211 amino-acid substitutions conferring resistance to ruxolitinib (INCB018424) and cross-resistance to the JAK2 inhibitors AZD1480, CYT-387 and lestaurtinib. In contrast, these resistant variants were fully sensitive to fedratinib. Structural modeling, coupled with mutagenesis and biochemical studies, revealed dual binding sites for fedratinib. *In vitro* binding assays using purified proteins showed strong affinity for the substrate-binding site (K_d_ = 20 nM) while affinity for the ATP site was poor (K_d_ = ~8 μM). Our studies demonstrate that mutations affecting the substrate-binding pocket encode a catalytically incompetent kinase, thereby preventing emergence of resistant variants. Most importantly, our data suggest that in order to develop resistance-free kinase inhibitors, the next-generation drug design should target the substrate-binding site.

Myeloproliferative neoplasms (MPNs) are a group of hematologic malignancies that include Ph^+^ chronic myeloid leukemia (CML) and Ph^−^ diseases that includes primary myelofibrosis (MF), polycythemia vera (PV), and essential thrombocythemia (ET). The discovery that constitutive ABL kinase activity is sufficient and necessary to cause CML laid the foundation for development of imatinib as a target-directed therapy^1^,^2^. The clinical success of BCR-ABL inhibitors for the treatment of CML not only revolutionized the anti-kinase therapy but also enforced the idea to identify the genetic lesions in other neoplastic diseases for therapeutic targeting^2^,^3^,^4^. In 2005, four groups reported kinase-activating mutations in JAK2 (JAK2-V617F) from BCR-ABL-negative MPN patients^5^,^6^,^7^,^8^. This discovery generated great interest in treating MPNs by targeting JAK2 with small-molecule kinase inhibitors.

JAK2 is a cytosolic tyrosine kinase activated by cytokine-mediated receptor dimerization, resulting in phosphorylation of STATs required for cell proliferation, survival and myeloid development, as well as for the initial stages of the immune response^9^. Constitutive JAK2 signaling has been implicated in many other cancers—such as myeloid malignancies, breast cancers and B-cell leukemias^10^ and lymphomas^11^. This provides a strong rationale for JAK2 targeting, and suggests that the resultant therapies would have broad therapeutic potential. As proof of concept, JAK2-V617F was expressed in mouse hematopoietic cells, generating a tractable mouse model of PV and MF^12^,^13^,^14^. In each of these disease models, treatment with small-molecule JAK2-kinase inhibitors induced apoptotic cell death and prolonged the survival of mice^13^,^15^,^16^,^17^. Collectively, these observations paved the way for clinical development of JAK2-targeted therapeutics.

The JAK2 inhibitor ruxolitinib was recently approved for the treatment of MF and PV, and numerous other inhibitors are in phase-II/-III clinical trials^18^. Ruxolitinib and other JAK2 inhibitors have shown significant improvement in quality of life. However, unlike other tyrosine kinase inhibitor (TKI) therapy, they do not have clonal selectivity^3^,^19^,^20^,^21^. Given that the therapeutic response to TKI therapy is mediated by oncogene addiction, clinical and mouse studies suggest that MPNs induced by JAK2-V617F are not addicted to the driver oncogene. Three principal mechanisms i.e. genetic streamlining, oncogenic shock and synthetic lethality govern addiction to the driver oncogene^22^,^23^. There are intensive efforts to develop combination therapies to achieve clonal selectivity for JAK2 inhibitors, perhaps by inducing synthetic lethality. In preclinical mouse models, combinations of ruxolitinib with inhibitors of PI3K, Hedgehog, HDAC, BCL2 and interferon–alpha have shown clonal selectivity for JAK2-V617^24^. Clinical trials are undergoing for these combinatorial treatments^24^. Given the prevalence of genetic resistance in response to anti-kinase therapy under selective pressure, we reasoned that genetic resistance to JAK2 inhibitors would emerge once treatment specific to the JAK2 mutant cells is established. Therefore, using JAK2-V617F-addicted cells we sought to understand patterns of resistance to JAK2 inhibitors, and to glean functional insights for further drug refinement.

We performed an unbiased chemical-genetic screen using two different JAK2 inhibitors, ruxolitinib and fedratinib, to identify a comprehensive set of drug-resistant variants, in order to glean regulatory mechanisms of resistance. Our screen identified 211 resistance mutations against ruxolitinib, but a complete lack of resistance against fedratinib. The resistance mutations conferred cross-resistance to other JAK2 inhibitors—AZD1480, CYT-387 and lestaurtinib, but failed to confer resistance against fedratinib. Biochemical characterization and structural modeling revealed that fedratinib simultaneously binds to both ATP-binding and peptide-substrate-binding sites, thereby preventing emergence of resistant clones.

## Results

### Lack of genetic resistance against fedratinib

**Figure 1:**
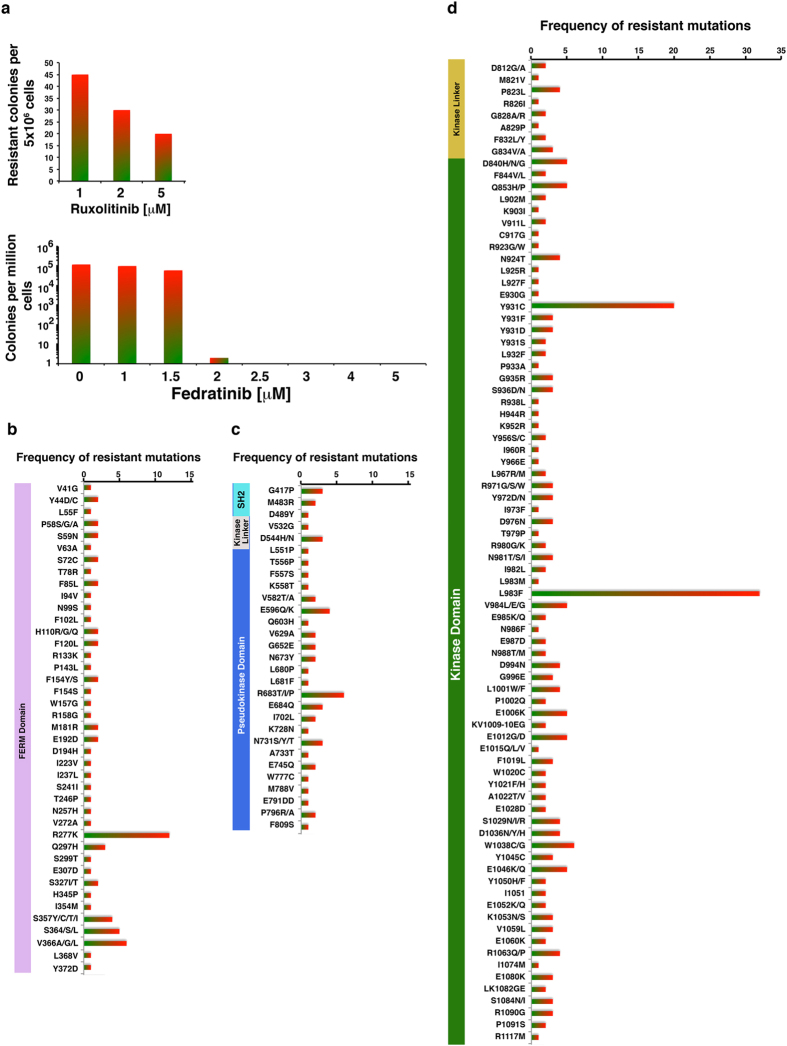
Random mutagenesis and *in vitro* drug-resistant screening reveal lack of genetic resistance against fedratinib. (**a**) Frequency of resistance of BaF3 cells transformed with randomly mutagenized JAK2-V617F. For each experiment, 1.0 × 10^7^ BaF3 cells were transduced with randomly mutagenized JAK2-V617F virus. After 16 to 20 hours, cells were exposed to different concentrations of drug inhibitors, for two weeks. The number of clones obtained per 5 × 10^6^ cells from two independent experiments was averaged and plotted. (**b**) Frequency of resistant mutations from the FERM domain. (**c**) Frequency of resistant mutations from the SH2 and pseudokinase (JH2) domains. (**d**) Frequency of resistant mutations from the kinase-linker and kinase (JH1) domains.

We performed a ruxolitinib resistant screen using BaF3-MPL cells that showed emergence of resistant clones (data not shown). Although these clones conferred robust resistance to ruxolitinib, sequencing did not reveal mutations. *In vitro* characterization of these clones showed both higher IC50 and increased resistance to ruxolitinib, thus suggesting that the BaF3-MPL cells expressing JAK2-V617F are not addicted to JAK2 because MPL overexpression seemingly bypasses the JAK2 dependent survival. Therefore, we performed screening using parental BaF3 cells transduced with randomly mutagenized JAK2-V617F and two clinically relevant JAK2 inhibitors: ruxolitinib and fedratinib. Ruxolitinib-resistant clones emerged at 1, 2 and 5 μM inhibitor—representing 10−, 25− and 50-fold increases in IC50 values for JAK2-V617F (~100 nM), respectively (Fig. 1a). In contrast, selection against fedratinib at concentrations 2-fold above IC50 (~0.9 μM) did not result in any resistant clones (Fig. 1a, lower panel). From the 190 ruxolitinib-resistant colonies, we identified 211 distinct amino-acid substitutions affecting 149 residues. Amino-acid substitutions at 58% of the positions were identified more than once (Fig. 1b–d).

**Figure 2:**
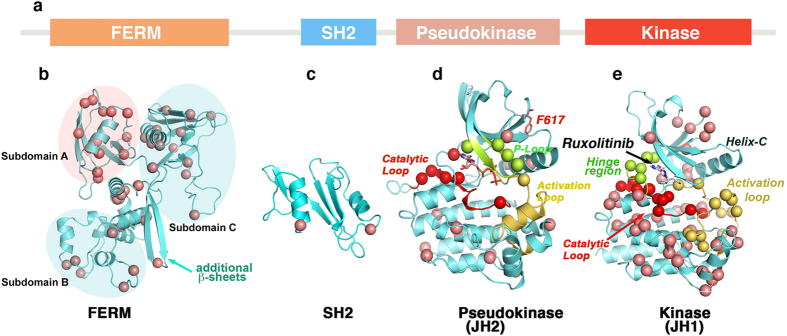
Mapping of ruxolitinib-resistant mutations on JAK2 structure. (**a**) Primary structure of JAK2 kinase showing structural subdomains. (**b**) FERM domain model, developed by homology modeling using FAK coordinates (PDB: 2J0J and 2J0L), showing different subdomains and distribution of resistant variants. Subdomains A and C have the highest number of mutations. In contrast to FERM domain of FAK, we identified two extra β-sheets in JAK2 between subdomains B and C (cyan arrow). (**c**) Homology model of the SH2 domain based on SRC and ABL structures (1OPK and 2SRC), showing resistant mutations in the α-helices. (**d**) Pseudokinase structure (PDB: 4FVR) showing the oncogenic mutation V617F (green) and many resistant variants clustered in the catalytic site—around the ATP-binding site (red), P-loop (green), and helix-C (pink) (**e**). JAK2 kinase domain structure (PDB: 2B7A) docked with ruxolitinib showing the distribution of resistant mutations. Many mutations are clustered around the ATP binding and catalytic sites, and in the C-lobe. The catalytic site is comprised of a catalytic loop (red), hinge region (green) and activation loop (yellow). α-C of mutant residues are presented as circles.

We next mapped resistant mutations onto crystal structures of JAK2 kinase domains^25^, pseudokinase domains^26^, and homology-based structural models of the FERM and SH2 domains, developed using SWISS-MODEL^27^ and the structures of FAK (PDB:2J0J, 2J0L and 2J0M), SRC (PDB:2SRC) and ABL kinases (PDB: 1OPJ, 1OPL, 2G2H and 2G2I) (Fig. 2). We identified four structural hot spots in the kinase catalytic domain—specifically in the active site and allosteric sites A, B and C (Supplementary Fig. 1). Interestingly, these hot spots are similar to imatinib-resistant mutations identified from both drug-resistant screens and relapsed patients^28^,^29^. These mutations are, therefore, likely to be involved in allosteric regulation of kinase conformation (probably by inter- and intra-domain interactions modulating auto and co-inhibitory state) and drug-binding state.

### Point mutations in FERM, SH2, pseudokinase, and kinase domains confer resistance

To verify the activity of individual mutations (since some resistant clones carried two or more mutations) and to elucidate the mechanisms of resistance, we generated 40 different variants in the FERM, SH2, JH2, and kinase domains of JAK2-V617F, using site-directed mutagenesis. Dose-response analysis revealed that all of these mutants are resistant to drug inhibition, except variant D976N (Supplementary Fig. 2, and Supplementary table 1). Three resistant variants—Y931C, R971G and L983F—demonstrated very high IC50 values (>10 fold), thus explaining their more frequent presence in the screen. Interestingly, many resistant variants showed higher IC50 values when the cells were grown in the absence of cytokine, presumably due to receptor free Jak2 signaling (i.e. JAK2 is not bound to cytokine receptor). It is a general consensus in the field that JAK2 is constitutively bound to its receptor but this model lacks any experimental validation. Recent studies suggest for the existence of an autoinhibited receptor-free form of Jak2^30^,^31^, which seems more plausible. To address this, we performed experiments to quantify the receptor-free form and bound form of Jak2 in the presence and absence of cytokines using two different protein crosslinkers. These experiments revealed that in the absence of receptor activation (absence of cytokine) a significant proportion of Jak2 is in free-form while addition of cytokine completely translocate them to the receptor (Supplementary Fig. 3).

In contrast, variants M181R, R971G, L983F, E1006K, E1046K, and R1063P showed higher resistance when the receptors were activated by exogenous cytokines (Supplementary Fig. 2); this suggests that these mutations affect the conformational state stabilized during receptor activation—leading to stabilization or destabilization of the active state, which directly correlates with kinase activity and cellular transformation potential, as demonstrated for imatinib-resistant mutations in BCR-ABL^28^,^32^. We therefore performed BaF3 cell transformation assays using the modified Whitlock-Witte assay that can distinguish subtle differences in kinase activation and cell transformation potential. Three variants (R683T, Y931C and Y931F) showed robust transformation, similar to BCR-ABL and TEL-JAK2. Many variants with mutations in the FERM domain (Y44C, P58A, N99S, H110R, R277K, and V366G) and SH2 domain (G417P and M483R) showed increased transformation potential (Supplementary Fig. 4A), suggesting that these variants stabilize an active kinase conformation, possibly by relieving the autoinhibitory constraints as proposed for activation of JAK3 kinase by FERM-domain mutations^33^. Resistant mutations in the pseudokinase domain showed either increased (R683T and G652E) or decreased (L511P, TF556PS, V582A, E596QS, L680P, E684Y, and N731) transformation potential (Supplementary Fig. 4A). Structural modeling studies of these mutations, using the recently crystallized pseudokinase coordinates, suggest that some variants affect the catalytic activity of JH2 (L551P, TF556PS, L680P, R683T and E684Q), while others (V582A, E596Q, G652E and N731Y) most likely affect JH2 autodimerization^26^. This suggests drug binding is altered and resistance conferred when gain in kinase activity of the pseudokinase domain destabilizes the conformation of JH1^26^,^34^ Likewise, mutations in the kinase domain showed either higher (L902M, Y931C, Y931F, and E1080K) or lower (D840N, N924T, L927F, Q853P, D976N, N981S, R971G, L1001W, and E1006K) transformation potentials (Supplementary Fig. 4B). In the absence of growth factor IL-3 (i.e., no receptor activation), hyper-activating mutations conferred greater resistance; interestingly, in the presence of IL-3, mutants that showed lower transformation potential (M181R, R971G, L983F and E1006K), also conferred greater resistance (Supplementary Figs 2, and Supplementary Table 1). This suggests that under normal conditions, Jak2 that is not associated with receptor is locked in an inactive state, and receptor binding through the FERM/JH2 domain relieves steric constraints, allowing the potential to be activated. In summary, mutations in allosteric sites of Jak2 alter inter-molecular interactions between FERM, SH2, and JH2 domains and cytokine receptors, which affects kinase conformation and, perhaps, drug-binding affinity.

### Active site variants confer resistance

**Figure 3:**
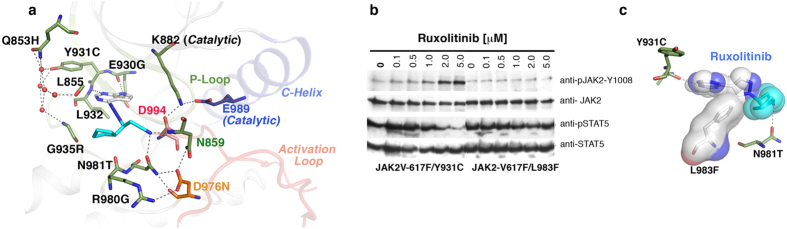
Resistant variants from the active site act by steric hindrance or disrupting active-site integrity. (**a**) A cartoon depiction of JAK2 active site bound with ruxolitinib (blue sticks) showing its interaction with Glu 930 and Asn 981 by hydrogen bonding (dashed line). Residue Tyr 931 interacts with Gln 853, Leu 855 and Gly 935 by hydrogen bonds with 5 water molecules (red circles). Residue Asp 994 (orange stick) from the DFG motif directly interacts with ruxolitinib and catalytic Lys 882. (**b**) Immunoblot analysis of BaF3 cells expressing JAK2-V617 variants Y931C and L983F, showing dose-dependent inhibition of Y931C by ruxolitinib (L983F is completely resistant). Upper panel showing increasing phospho-JAK2 levels with increasing drug concentration, possibly due to preferential binding and stabilization of the active conformation^73^. Lower panel showing phospho-STAT5 level. Blots were stripped and reprobed for total JAK2 and STAT5. (**c**) Structural model of ruxolitinib, showing steric clash with phenylalanine substituted for Leu 983.

Mutations within the ATP-binding site usually confer resistance by directly blocking drug binding, rather than by modulating conformational dynamics (the latter is a common mechanism used by allosteric mutations). Accordingly, many mutations clustered in the active site (i.e., Y966E, L983F, N986Y, E987D, and E1046K) were resistant, but did not exhibit any change in IC50 values when assayed in the presence or absence of IL-3 (Supplementary Fig. 2), or in JAK2-V617F malignant potential (Supplementary Fig. 4). This implies that these mutations were directly impacting drug binding, rather than modulating the kinase dynamics by altering inter- or intramolecular interactions. We next performed molecular-docking of ruxolitinib, by targeted docking to the ATP-site and blind docking to the whole kinase domain by SwissDock^35^ using the JAK2 coordinate (PDB:2B7A) . These analyses revealed that ruxolitinib anchors to the ATP binding cleft by three hydrogen bonds where it forms two hydrogen bonds in the hinge region with residues Glu 930, Leu 932 and one in catalytic site with Asn 981. Ruxolitinib requires DFG-in conformation for binding (Fig. 3a)—as seen for other type-1 kinase inhibitors. The hydrophobic rings of ruxolitinib make van der Walls interactions with the side chains of residues Val 911, Met 929, Leu 855, Lys 882, Leu932, Pro 933, Gly 935, Arg 980, Asn 981, Leu 983, and Asp 994 (Supplementary Fig. 5). Structural modeling of the active-site mutations suggests three principal mechanisms governing resistance: **(i)**, loss of hydrogen-bond for anchoring, by substituting threonine for Asn 981, while variants E930G and L932F may disrupt hydrogen bond interactions perhaps by altering the dynamics of hinge region (Fig. 3a and Supplementary Fig. 5); **(ii)** steric hindrance (eg, V911L, L927F, G935R, and L983F) (Fig. 3c); and **(iii)** active-site destabilization, by mutations in the kinase hinge (Y931C, E930G, L932F, P933A, and E985K), the catalytic loop (N981I/T) and the DFG motif (D994N) (Supplementary Figs 5–7). Amongst these, Tyr 931 coordinates the structural integrity of the P-loop and active site, by interacting through four water molecules with Gln 853, Leu 855 and Gly 935 (Fig. 3a). In cellular transformation assays, cysteine or phenylalanine substitution at this position disrupted these interactions, resulting in full activation of the kinase much like BCR-ABL and TEL-JAK2 (Supplementary Fig. 7). Structural modeling of activation-loop variants (R971G, L1001W and E1006K), suggests that these mutations should destabilize the activation loop from the active kinase conformation (Supplementary Fig. 8A–C), suggesting that ruxolitinib preferably binds an active kinase conformation. The fact that phospho-JAK2 levels increase with increasing inhibitor concentration (Fig. 3b), also suggests that ruxolitinib binds the active state. If this model is correct, then activating variants of JAK2 should be hypersensitive to ruxolitinib inhibition, rather than conferring resistance. In contrast, we observed that activating variants R683T and Y931C conferred resistance to ruxolitinib (Fig. 3b, and Supplementary Fig. 2). Likewise, fully activated JAK2 (TEL-JAK2) exhibited 4-fold higher resistance than JAK2-V617F (Supplementary Fig. 8D), suggesting that ruxolitinib has reduced affinity for fully activated kinase and, instead, preferentially binds an intermediate active-state kinase conformation, much as intermediate kinase conformations of ABL and SRC that adopt DFG-in conformation, but without fully extended activation loops^36^.

### Ruxolitinib-resistant mutations confer resistance across a panel of JAK inhibitors

**Figure 4:**
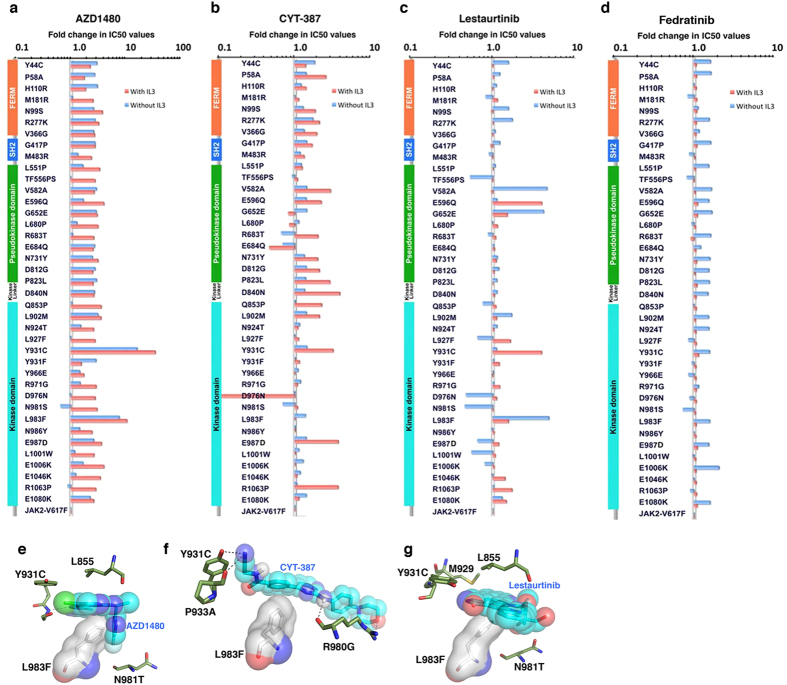
Ruxolitinib-resistant variants confer cross resistance to AZD1480, CYT-387 and lestaurtinib—but not to fedratinib. IC50 values for AZD1480 (**a**), CYT-387 (**b**), lestaurtinib (**c**) and fedratinib (**d**) were normalized to 1 with JAK2-V617F and plotted on a semilogarithmic scale. Structural domains, FERM, SH2 and pseudokinase, are indicated to the left side of each graph. The most frequent and highly resistant mutation, L983F, is sensitive to CYT-387 and fedratinib. Ruxolitinib-resistant variants are fully sensitive or mildly resistant to TG10138, when cells were grown with or without IL-3, respectively. (**e**) Structural model of AZD1480 bound to ATP binding site, showing steric clash with phenylalanine substituted for Leu 983. (**f**) Structural model of CYT-387 bound to ATP binding site, showing no direct clash with bulkier phenylalanine substitution for Leu 983, explains why this variant is still sensitive to CYT-387. (**g**) Structural model of lestaurtinib bound to the ATP-binding site, showing steric clash with phenylalanine substituted for leucine 983.

Because each inhibitor binds a unique conformational state, engaging different sets of amino acid residues^37^, we assumed that some ruxolitinib-resistant variants might be sensitive to other JAK2 inhibitors. We therefore carried out dose-response analyses with ruxolitinib-resistant variants and four other JAK2 inhibitors that are in clinical trials (AZD1840, CYT-387, lestaurtinib and fedratinib). Of these, many mutants displayed cross-resistance to AZD1840, CYT-387 and Lestaurtinib—but not to fedratinib (Fig. 4a–d). As expected, drug response varied with mutation type and cytokine receptor activation, enforcing the notion that each inhibitor targets distinct kinase conformations. For instance, Y931C is fully sensitive to lestaurtinib in the absence of IL-3, but showed 5-fold resistance in the presence of IL-3 (Fig. 4c). Likewise, while pseudokinase domain variants showed greater resistance to lestaurtinib in the absence of IL-3, and greater resistance to CYT-387 in the presence of IL-3; FERM domain variants also demonstrated greater resistance to CYT-387 in the presence of IL-3 (Fig. 4). Finally, variant L983F showed resistance to AZD1480 and lestaurtinib, but retained full sensitivity to CYT-387. Structural modeling and docking revealed that AZD1480, lestaurtinib and CYT-387 are type-I ATP-site inhibitors (Fig. 4e–g and supplementary Figs 9–11). Substitution of Phe for Leu 983 caused steric hindrance to AZD1480 and lestaurtinib, but did not affect CYT-387 binding (Fig. 4e–g) providing an explanation for the sensitivity of L983F variant to CYT-387. These data suggest that each inhibitor targets a distinct kinase conformation, and alteration in conformational dynamics by allosteric mutations destabilize drug binding state, thus conferring resistance^38^. Once the ternary structure of JAK2 is solved, our mutational data will be instrumental in understanding how inter and intramolecular interactions modulate kinase conformation and activation.

### Fedratinib binds both ATP and peptide substrate-binding sites

All ruxolitinib-resistant JAK2 variants showed a complete lack of resistance to fedratinib, particularly when receptor was activated by IL-3 (Fig. 4d). This suggests that fedratinib binds either a receptor-bound form or a fully activated kinase. To distinguish these possibilities, we performed a dose-response analysis using receptor-free fully activated JAK2 (TEL-JAK2), and found a ~3-fold increase in hypersensitivity to fedratinib in comparison to JAK2-V617F (data not shown)—suggesting that fedratinib specifically targets the fully active kinase conformation. To rule out off-target effects of fedratinib on BaF3 proliferation, we used two different approaches: (1) overexpression of non-kinase targets (eg, constitutively activated STAT5 or BCL2), or kinase targets known to have lower affinity for fedratinib (eg, JAK1 or JAK3); and (2) heterodimeric expression of JAK2 with JAK1 and JAK3, as JAK1-JAK2 heterodimers confer resistance^39^. While all of these cell lines had increased IC50 values, JAK1 conferred the greatest resistance (Supplementary Fig. 12a–c). IC50 values for phospho-STAT5 in JAK1- and JAK3-transduced cells were greater than 10 and 5 μM, respectively (Supplementary Fig. 12d). To rule out off-target effects in our resistant screens, we performed soft-agar colony assays using JAK1- and JAK3-expressing BaF3 cells; TEL-JAK1 and TEL-JAK3 supported colony growth up to 6 and 4 μM of fedratinib, respectively (Supplementary Fig. 12e). However, under similar conditions, the JAK2-V617F and ruxolitinib resistant variant, JAK2-V617F-Y931C, failed to grow above 3 μM of fedratinib (Supplementary Fig. 12e). Thus, inhibition of JAK2 and its resistant variants is not due to off-target effects.

**Figure 5:**
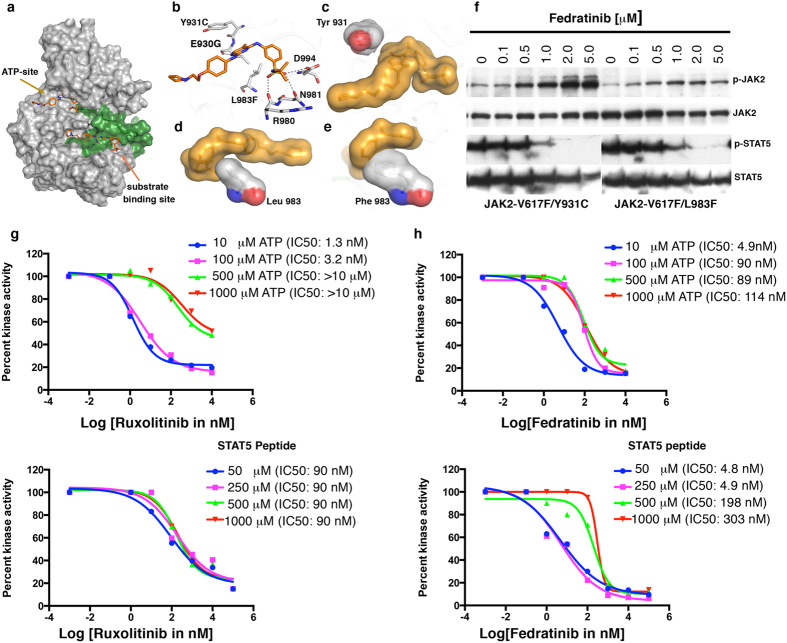
Fedratinib binds to ATP and substrate-binding sites in JAK2 kinase. (**a**) Surface depiction of JAK2 kinase in active conformation, showing activation loop (green), fedratinib binding (orange stick) to ATP-binding and substrate-binding sites (arrows). (**b**) Enlarged view of ATP-binding site, showing fedratinib anchorage by hydrogen bonding (dashed gray line) with Glu 930 (hinge region), Arg 980 and Asn 981 (catalytic site), and Asp 994 of the DFG motif. (**c**) Surface depiction of Tyr 931showing no direct interaction with fedratinib. However, resistant variant Y931C confers moderate resistance, possibly by modulating the architecture of the active site as a result of loss of hydrogen bonding with water molecules (Fig. 2a). (**d**) Surface depiction of Leu 983 from catalytic site. (**e**) Effect of phenylalanine substitution for Leu 983 on fedratinib binding and resistance. (**f**) Immunoblot analysis of BaF3 cells expressing JAK2-V617 variants Y931C and L983F, showing dose-dependent inhibition by fedratinib. Upper and lower panels show increasing phospho-JAK2 and decreasing phospho-STAT5, respectively, with increasing drug concentrations. Blots were stripped and reprobed for total JAK2 and STAT5 levels. (**g**) Steady-state kinetic analysis of purified JAK2 kinase inhibition by INCB01842 with increasing ATP concentrations (upper panel) and STAT5 peptide-substrate concentrations (lower panel). IC50 values for kinase inhibition are shown in parentheses against each concentration. (**h**) Purified JAK2 kinase inhibition by fedratinib, showing a ~20-fold change in IC50 value (upper panel) with increasing ATP concentration. Similarly, increasing substrate concentrations (lower panel) showed an even higher shift in IC50 value (~60 fold).

To better understand the molecular mechanism of fedratinib inhibition, we performed fedratinib molecular-docking experiments, by targeted docking to the ATP-site, substrate site and blind docking to the whole kinase domain (PDB:2B7A) by SWissDock^35^. These docking analyses revealed that fedratinib can simultaneously bind to two different sites in the kinase domain, the ATP site and the peptide substrate-binding site (Fig. 5a). Interestingly, dual binding of a kinase inhibitor with a similar chemical scaffold has been described previously (Supplementary Fig. 13)^40^. In order to validate the dual binding we further performed additional docking on another platform (AutoDock) and molecular dynamic simulations (MDS). Simulations using Autodock predict that fedratinib binds preferentially to the substrate-binding site. At the same time, in agreement with known experimental data, ruxolitinib is predicted to bind to the ATP binding site. Preferential binding of fedratinib to the substrate-binding pocket is indicated by both the predicted binding energies and the relative paucity of contacts unique to ATP binding site, i.e., those involving residues 930–940 (Supplementary Fig. 14 and supplementary table 2). This is in contrast to results obtained for ruxolitinib, which shows strong preference for the ATP binding site even when using the large simulation box that enables sampling both pockets (Supplementary Fig. 15 and supplementary table 3). In addition, predicted binding energies for the two compounds and the two alternative binding sites are also consistent with preferential binding of fedratinib to the substrate-binding site. The results shown in supplementary Figs 14 and 15 were obtained using 2B7A as the structure of the protein. Similar results were obtained also using 3TJD (data not shown). Molecular dynamics simulations provide further support that fedratinib binds at the substrate-binding site. The ligand remains bound to the protein in the course of 50 ns simulations, starting from an initial docking pose predicted by AutoDock (Supplementary Fig. 16A). Moreover, while both protein and ligand fluctuate, a pattern of relatively stable hydrogen bond was observed with Glu 1016 within the substrate-binding pocket (Supplementary figure 16B).

To determine whether fedratinib inhibits by binding the ATP site or the peptide substrate-site, we performed structural modeling of two resistant ATP-site variants, Y931C and L983F, (Fig. 5b–e). These variants conferred mild resistance (~1.5 fold change in IC50 values) in cellular and biochemical assays, when cells were grown without IL-3 (Figs 4d and 5f). However, this resistance was abolished when receptors were activated by IL-3. This suggests that fedratinib binds either the substrate-binding site or stabilizes receptor-bound kinase, and it’s binding to the second site masks the resistance mediated by the ATP-site mutations. To confirm fedratinib binding to both ATP and substrate-binding sites, we performed a steady-state enzyme kinetic assay, using purified JAK2 kinase domain with increasing ATP and peptide substrate (STAT5). Ruxolitinib shifted IC50 values only with increasing ATP concentrations, confirming that it is an ATP-competitive inhibitor (Fig. 5g). In contrast, fedratinib shifted IC50 values with both increasing ATP and substrate concentrations (Fig. 5h), thus, validating the *in silico* docking observation. These experiments demonstrated that ruxolitinib binds only the ATP site, while fedratinib binds both ATP and substrate-binding sites.

### Fedratinib binds to substrate-binding site with stronger affinity than the ATP site

**Figure 6:**
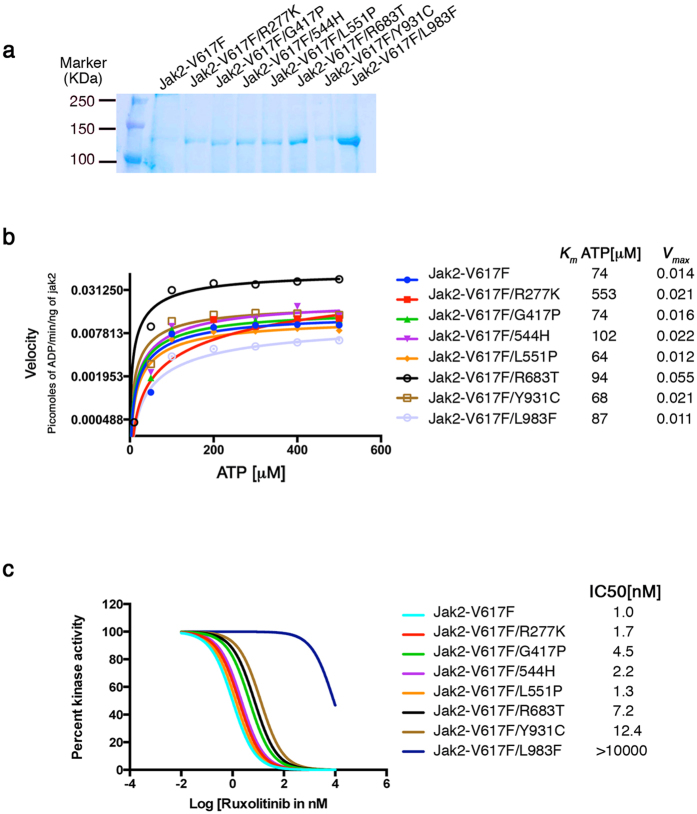
Allosteric ruxolitinib resistant variants activate the enzymatic activity. (**a**) A coomassie blue stained gel showing partially purified full length JAK2 and its variants. (**b**) Steady state enzyme kinetics showing the activation of ruxolitinib resistant mutants except L983F. Variant R683T is highly active, while Y931C showed increased V_max_ and and reduced K_m(ATP)_. The Apparent values for K_m(ATP)_ and V_max_ are shown on the right side of the graph for each mutant. (**c**) Dose response analysis against ruxolitinib showing resistance to inhibition. IC50 values for each mutant (as an indirect measure for drug affinity) are indicated on the right side of the graph.

To understand the mechanisms underlying how mutations from the FERM, SH2 and pseudokinase domain confer resistance, we performed steady state kinetics to determine the Km for ATP and V_max_. One principal mechanism for developing resistance against ATP-competitive inhibitors is to select for kinase activating mutations. In some instances these activating mutations may change the Km for ATP providing additional support to compete out inhibitor binding; a common mechanism exploited by gatekeeper mutations to confer resistance. To determine the kinetic parameters we expressed full length JAK2-V617F and seven ruxolitinib resistant variants in insect cells, proteins were purified using Strep-Tactin resins (Fig. 6a). Kinetic experiments were carried out at saturating concentration of STAT5 peptide (50 uM) with varying concentrations of ATP to determine the K_m (ATP)_^app^ and V_max_ (Fig. 6b). These analyses revealed that allosteric mutations have higher enzymatic activity as anticipated from the cell based transformation assay (Supplementary Fig. 3A,B). Resistant variant R683T (pseudokinase domain) displayed a 5-fold increase in V_max_, while variants R277K (FERM domain), G417P (SH2 domain), D544H (SH2-pseudokinase linker) and Y931C (Kinase domain) displayed ~2 fold increase V_max_ compared to JAK2-V617F. Interestingly variants L551P (pseudokinase domain) and Y931C (kinase domain) have reduced K_m (ATP)_^app^ . However, resistant variant L983F did not show any significant change in K_m (ATP)_^app^ and V_max_. Whereas, a kinetic analysis of purified kinase domain displayed ~20 fold (Y931C), 4 fold (E930G) and 2 fold (L983F) increase in V_max_ with significant changes in K_m (ATP)_^app^ by Y931C and L983F (Supplementary Fig. 16) . The ATP K_m_ value for full length JAK2-V617F and kinase domain are in close agreement as recently reported^41^. Dose response analysis against ruxolitinib, as an indirect measure of affinity, using these full-length proteins showed resistance to ruxolitinib, however, with differences in IC50 values reflecting their apparent differences in mediating resistance (Fig. 6c). Likewise, purified kinase domains having ruxolitinib resistant mutations conferred resistance to ruxolitinib but not to fedratinib (Supplementary Fig. 16C,D). However, dose response analysis against CYT-387 showed resistance for Y931C but both E930G and L983F were sensitive (Supplementary Fig. 16e).

**Figure 7:**
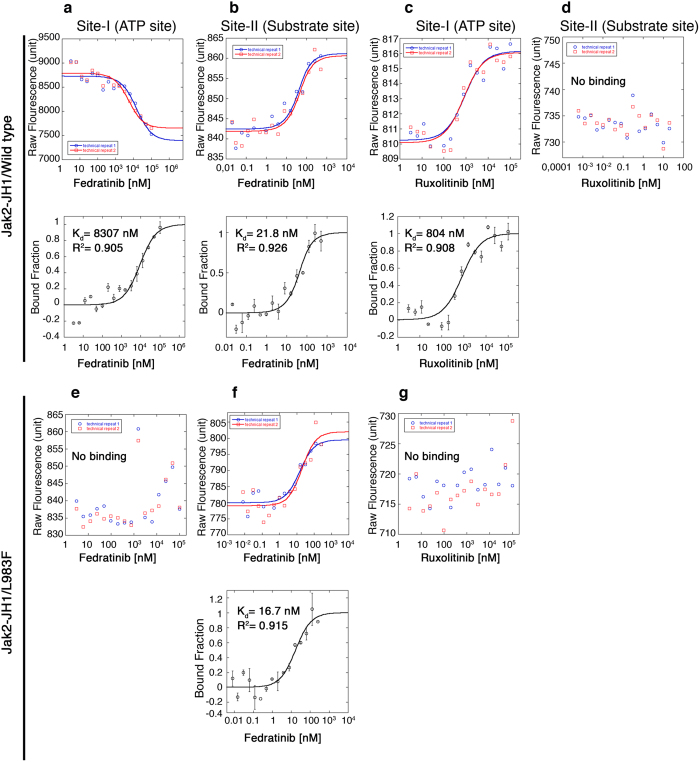
Fedratinib binds to substrate-site with higher affinity than ATP site. (**a**,**b**) Microscale thermophoresis (MST) analysis showing the dual binding of fedratinib to JAK2-WT. Top panels are showing the fluorescence. Bottom panels are showing the bound fractions of inhibitors and proteins to calculate the dissociation constant (Kd), which is mentioned in the parenthesis. Fedratinib binding was carried out with label free proteins. (**c**,**d**) MST analysis shows a single binding site (Site I-ATP site) for ruxolitinib with JAK2-WT. Top panels are showing the fluorescence. Bottom panels are showing the bound fractions of inhibitors and proteins to calculate the dissociation constant (Kd), which is mentioned in the parenthesis. Because, ruxolitinib showed auto-fluorescence that precluded label free binding analysis, we performed ruxolitinib binding with fluorescently labeled proteins. Note, we could not detect any secondary binding site for ruxolitinib. (**e**,**f**) MST analysis with JAK2-L983F showed only one binding site for fedratinib at site –II (substrate site). A phenylalanine substitution for Leu 983 causes steric hindrance to fedratinib and it no longer can bind to site-I, ATP site. In contrast, substrate site is not affected by this mutation and therefore binding of fedratinib to site-II is not affected showing similar *kd* values. Top panels are showing the fluorescence. Bottom panels are showing the bound fractions of inhibitors and proteins to calculate the dissociation constant (Kd), which is mentioned in the parenthesis. (**g**) MST analysis with JAK2-L983F showing loss of ruxolitinib binding with mutant L983F. These experiments were performed twice in duplicates.

To further characterize the dual binding of fedratinib we performed biophysical measurements using purified kinase domains of JAK2-WT and JAK2-L983F by Microscale Thermophoresis (MST). Thermophoresis measures the motion of molecules in microscopic temperature gradients that allows measuring the affinity constants and binding stoichiometry of a wide variety of interactions in the binding equilibrium. We reasoned that if fedratinib binds to two different sites i. e. the ATP and the substrate site, the use of an ATP site mutant for binding analysis will show only one binding site. As predicted, fedratinib showed two binding sites in JAK2-WT with binding affinities having Kd values of 8307 nM (Site –I/ATP site) and 21 nM (Site-II/substrate site), Fig. 7a,b. A similar analysis with ruxolitinib showed only one binding site (ATP site) having Kd of 804 nM, Fig. 7c,d. Perhaps more striking, ATP site variant JAK2-L983F showed only one binding site, site-II/substrate site, having a Kd value of 17 nM (Fig. 7e,f). In contrast, ruxolitinib no longer binds to JAK2-L983F (Fig. 7g). These data confirms *in silico* docking simulations, which predicted fedratinib binds to substrate site with higher affinity than ATP site.

### Mutations in the ATP and substrate-binding sites confer moderate resistance to fedratinib

**Figure 8:**
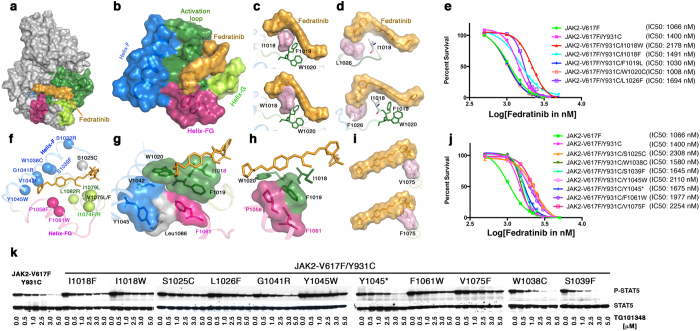
Mutagenesis of residues in the substrate-binding pocket confers moderate resistance to fedratinib. (**a**) Surface depiction of JAK2 kinase in active conformation, showing the activation loop (green) and binding of fedratinib (yellow) in the substrate-binding pocket. (**b**) Substrate-binding pocket is comprised of an activation loop (green) and helices F (blue), FG (purple) and G (green). Amino-acid residues IFWY in the activation loop is a highly conserved kinase motif that serves as a hydrophobic platform for inhibitor and substrate-binding (green surface sandwiched between inhibitor and helix F). (**c**) Surface depiction of the IFWY motif (upper panel); an Ile 1018-to-tryptophan substitution results in steric clash with fedratinib (lower panel). (**d**) Surface depiction of Leu 1026 (upper panel); a Leu 1026-to-phenylalanine substitution results in steric clash with the benzene-sulfonamide group of fedratinib. (**e**) Dose-response cell proliferations showing moderate increase in IC50 values by JAK2 variants JAK2V617F-Y931C/I1018W and JAK2V617F-Y931C/L1026F; on the other hand, mutations at F1019L and W1020C resulted in inactive kinase (i.e., no change in IC50 values). (**f**) Mutations identified from drug resistant screen at low dose of fedratinib (2.5 μM) using BaF3 cells expressing JAK2-V617F/Y931C. The α-carbon of each resistant mutation is shown as a circle. (**g**) Surface depiction of Tyr 1045 showing the π-stacking with residues Val 1042 (helix-F), Leu1086 (helix H), Phe1061 (helix FG), and the IFWY activation-loop motif. A bulkier substitution at Y1045 would push the IFWY motif towards the inhibitor, thereby possibly conferring resistance. (**h**) A bulkier substitution at Pro 1058 would push the Phe 1019 of IFWY, which might affect fedratinib binding. (**i**) A bulkier substitution at Val1075 (e.g., to phenylalanine) would cause steric hindrance to fedratinib. (**j**) Dose-response growth curves showing higher IC50 values for S1025C, Y1045W, F1061W, and V1075F. Variants W1038C, S1039F and Y1045* encode weekend kinase (Fig. S14), but displayed subtle increases in IC50. (**k**) Immunoblot analysis of phospho STAT5 (upper panel) and total STAT5 (lower panel) in BaF3 cells expressing JAK2-V617/Y931C variants. Variants S1025C, Y1045W and V1075F are resistant to 5 μM fedratinib, and also conferred highest resistance in cell-proliferation assays.

To further probe the mechanism of fedratinib binding to the substrate-binding site, we created substrate-site mutations in the background of JAK2-V617F-Y931C by introducing bulkier, hydrophobic amino acids proximal to the inhibitor-binding site. We used the JAK2-V617F-Y931C, because blocking one binding site is probably insufficient to observe resistance conferred by a secondary site. As expected, mutations I1018F/W and L1026F reduced kinase activity, while F1019L, W1020C and Y1021H resulted in inactive kinase (Supplementary Fig. 17). I1018F/W and L1026F exhibited increased IC50 values in a dose-dependent cell proliferation assay (Fig. 8a–e) and a phospho-STAT5 inhibition assay (Fig. 8k). Next, we performed a resistant screen using 100 million BaF3 cells expressing JAK2-V617F-Y931C; cells were treated with fedratinib at 2.0 μM—with the assumption that a lower drug concentration may select for clones conferring mild resistance, which could inform the secondary binding site. This screen produced one resistant clone, sequencing of which revealed a total of 13 different mutations—all in the substrate-binding pocket (Fig. 8f). Structural modeling suggested that these mutations affect fedratinib binding either by direct steric hindrance (S1025C, S1039F, G1041R, V1042F, I1074F, and V1075F) or by affecting stability of the conserved IFWY motif of the activation loop in the substrate-binding site (W1038C, Y1045W/*, P1058F, F1061W, I1079L, and L1082R) (Fig. 8g–i). To validate these findings, we designed the mutations (S1025C, W1038C, S1039F, Y1045W/*, F1061W, V1075F) in the context of JAK2V617F-Y931C. As expected, all 13 variants displayed weaker kinase activity, compared to parental, JAK2-V617F/Y931C kinase (Supplementary Fig. 17). While 4 variants (S1025C, Y1045W, F1061W and V1075F) conferred strong biochemical and cellular resistance (Fig. 8J,K), others (W1038C, S1039F and Y1045*) conferred subtle changes in cellular IC50 but no change in STAT5 inhibition. This suggests either these variants are false positives or express a catalytically inactive kinase that acts as a sink for inhibitor, which is reflected in moderate change in drug response.

This study provides clear evidence for fedratinib binding to the substrate-binding pocket of JAK2 kinase. The data also suggest that dual binding of fedratinib—to both ATP and substrate-binding sites—prevents the emergence of resistant clones. These results help explain why fedratinib-mediated inhibition is greater when receptor is activated by cytokines—since growth factor-mediated receptor dimerization activates and stabilizes an active kinase conformation that allows easy access to the substrate-binding site. Altogether, these data suggest that mutations in the substrate-binding site would negatively affect the catalytic efficiency that will result in weak proliferation compared to normal cells. Thus these clones will not survive long, and, therefore, will be unable to confer resistance.

### JAK2-V617F/L983F confers *in vivo* resistance to ruxolitinib

**Figure 9:**
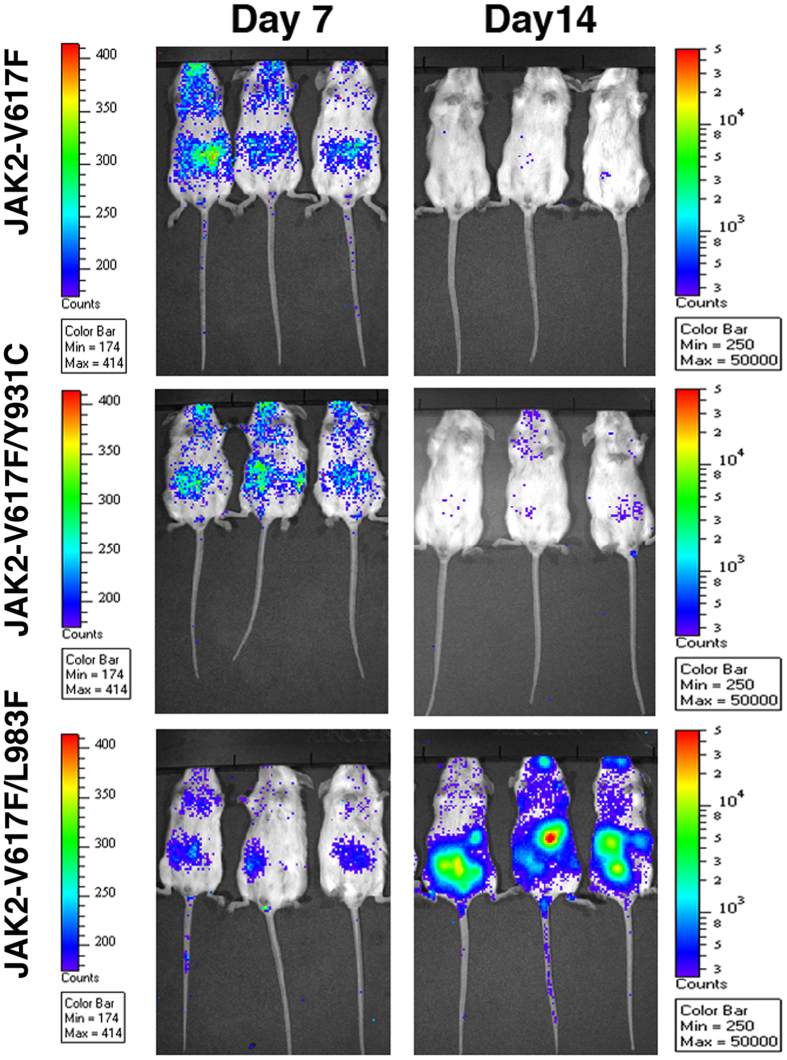
JAK2-V617F/L983F confers resistance *in vivo*. One million BaF3 cells expressing luciferase and a single JAK2-V617F variant were transplanted via tail vein in syngeneic Balb/c mice. After 3 days, mice were injected twice daily with ruxolitinib (100 mg/kg) for two weeks. Leukemic burden were measured by monitoring the bioluminiscence—7 days (left panel) and 14 days (right panel). As expected, JAK2-V617F/L983F conferred resistance, JAK2-V617F and JAK2-V617F/Y931C are responsive to ruxolitinib treatment.

The two most frequent mutations from our *in vitro* screen (i.e., Y931C and L983F) conferred high-grade resistance to all tested ATP-site inhibitors. To test whether they confer resistance *in vivo*, as a proof of concept, we transplanted Balb/c mice with 1 million BaF3 cells expressing luciferase and JAK2-V617F variants. As expected, twice daily administration of 100 mg/kg ruxolitinib eradicated the JAK2-V617F- and JAK2-V617F/Y931C-expressing cells (Fig. 9). On the other hand, a similar treatment was ineffective against JAK2-V617F/L983F (Fig. 9), thus demonstrating that this mutation confers resistance *in vivo*.

## Discussion

Resistance as a result of gene mutation highlights the limitations of targeted mono-therapy against the oncogenic kinases, and represents the next challenge in the development of ever more successful cancer chemotherapy. As is evident from previous work with BCR-ABL kinase inhibitors for chronic myeloid leukemia and in other kinase-targeted therapies, understanding mechanisms of drug resistance is a crucial first step in developing strategies to prevent or overcome it^42^,^43^. A recent approval of ruxolitinib by FDA for the treatment of MF prompted us to understand the patterns of resistance for JAK2 inhibitors that will be clinically problematic. Four different studies have performed resistant screening against JAK2 inhibitors using BaF3 cells transduced with either EPO or MPL receptors to facilitate JAK2-V617F dependence^39^,^44^,^45^,^46^. Only eight resistant mutations limited to kinase domain were identified from these screens, although numerous resistant clones developed that lacked mutations, presumably due to emergence of false-positives. One potential interpretation of these findings is that loss of JAK2 dependence occurs as a consequence of overexpression of cytokine receptors. Interestingly, an alternative explanation for the loss of Jak2 addiction has been proposed to be due to the heterodimerization of Jak kinases^39^. Therefore to avoid loss of JAK2 dependence we carried out resistant screening in parental BaF3 cells and showed robust selection of resistant clones against ruxolitinib with all of these clones showing the presence of resistant mutations.

Our results provide a comprehensive catalog of potential drug-resistant mutations against ruxolitinib and illuminate novel mechanisms by which alterations in the JAK2 structure thwart drug efficacy. In addition to previously reported mutations that so far have been limited to kinase domain, we identified novel substitutions of residues within and beyond the kinase domain that influence the conformational state of the kinase and confer drug resistance through allosteric mechanisms. Mutations in the kinase domain confer resistance using four different mechanisms. First, mutations conferring steric hindrance to drug; second, loss of hydrogen bonding for anchorage to the active site; third, destabilization of the active site and fourth, allosteric modulation of kinase conformation by FERM, SH2 and pseudokinase domain. An important principle to emerge from our *in vitro* screen and subsequent structural modeling is that numerous drug-resistant variants from the kinase domain destabilize an intermediate SRC like conformation of the JAK2 kinase to which ruxolitinib seemingly preferentially binds, thus shifting the equilibrium either toward the active or inactive kinase conformation that precludes drug binding. As our in silico data suggest that the ruxolitinib binds to intermediate-active kinase conformation that will stabilize the activation loop in open conformation, which in turn is stabilized by phosphorylation. Immunobloting of pJAK2 support this model where we observed a dose dependent increment in pJAK2 level upon ruxolitinib exposure. Many kinases show reduced phosphorylation when inhibited by small molecule inhibitors such as ABL, EGFR, SRC, PDGFR^32^. Nonetheless, erloitinb in EGFR and dasatinib in SRC/ABL stabilize the kinase in active conformation but these kinases do not show increased phosphorylation. These observations suggest two possibilities. First, phosphatase responsible for dephosphorylating the Jak2 is unable to bind intermediate-conformation stabilized by ruxolitinib. Second, the given phosphatase is directly or indirectly inhibited by ruxolitinib. In both scenarios, Jak2 would show a dose dependent increment in p-Jak2 levels with reduced p-STAT5. To support this notion, most allosteric MEK1/2 inhibitors show a dose dependent increment in p-MEK levels and stabilize the active phosphorylated conformation of the kinase. Besides, ruxolitinib induced stabilization of p-Jak2 conformation may activate the compensatory mechanisms (as Jak2 is tightly regulated by many signaling proteins such as SHP1, SHP2 and SOCS^47^) that may directly or indirectly affect the p-STAT5 levels. Perhaps a comprehensive proteomic studies with Jak2 inhibitors may resolve the mechanism. Nonetheless, these conformational dynamics are further modulated by growth factor binding to their receptors that directly affect both transformation potential and therapeutic response to drug inhibition (Supplementary Figs 2,3 and 4).

While the complete structure of JAK2 or its family members has still not been elucidated, the crystal structures of SRC, ABL and FAK kinases indicate that intramolecular interactions between the catalytic domain with pseudokinase and FERM domains are of major importance in regulating the dynamics and catalytic activity. By analogy with FAK kinases, in the autoinhibited state the kinase is locked in an inactive conformation where FERM domain and the JH1 and JH2 domains of JAK2 are tightly folded together. This prevents the JAK2 catalytic domain from being active by sequestering the activating tyrosine-phosphorylation from the activation loop implying that release of the FERM domain is an initiating step in kinase activation. In this regard, mutations from our screen are clustered in subdomains a, and b of the FERM domain that mediate direct contact with the kinase domain, which has been shown to be important for FAK activation. Interestingly all of these mutations have shown higher transformation potential, except M181R, and conferred resistance, suggesting these mutations relieve FERM mediated autoinhibition resulting in kinase activation and modulation of kinase conformation. In addition, FERM domain in JAK3 has also been shown to stabilize the active kinase conformation by direct physical interaction^33^. Although the nature of this interaction is not clear, it is tempting to speculate that mutant M181R (representing subdomain c) may be involved in stabilization of active kinase conformation. These observations present a simplistic model where subdomains a and b stabilize the autoinhibited state while subdomain c stabilize the active state, therefore mutations at interacting interfaces would affect kinase conformational state that may alter the affinity for drug binding. Likewise, the pseudokinase domain has been implicated in stabilizing the inhibitory and stimulatory interactions that regulate JH1 activity. However, the exact nature of the JH2-mediated inhibitory and stimulatory interactions that regulate kinase activity and modulation of kinase conformation is not known. We speculate that resistant mutations from the JH2 domain stabilize the inhibitory interactions as opposed to hyper-stabilization of the stimulatory interactions proposed for MPN mutations, V617F and R683T, because mutations from the pseudokinase domain conferred greater resistance in the absence of stimulatory signal when the receptors were not activated by growth factors (Supplementary Fig. 2). However, we do not know how these interactions are made, therefore further structural and biochemical studies are warranted to characterize the requisite molecular interactions. Finally, refinement in the structure of the FERM, SH2 and JH2 domain interactions with receptor and kinase domain is needed to explain how drug-resistant variants modulate the kinase conformation.

Emergence of resistant mutations highlights the fact that leukemic cells continue to depend on kinase activation for disease pathogenesis. Given that each inhibitor targets a unique conformation implies that some ruxolitinib resistance mutations might be sensitive to other JAK2 inhibitors. Our study demonstrates that, like ruxolitinib, other ATP site inhibitors (AZD1480, CYT-387 and lestaurtinib) are susceptible to resistance. However, in contrast, fedratinib showed a complete lack of genetic resistance due to its unique binding to both ATP and substrate-binding sites. Enzyme kinetics, *in vitro* binding and emergence of resistance mutations from the substrate-binding pocket demonstrate that fedratinib binds to both ATP and substrate-binding sites, with binding to the substrate-binding site showing higher affinity. It is possible that fedratinib’s ability to bind to the substrate-binding site prevents the emergence of resistance due to a counterselection for mutations in the substrate-binding site that would encode a weakened kinase. As a proof of concept, we performed a resistance screen using a substrate site BCR-ABL inhibitor, ONO12380^48^. As expected, ONO12380 suppressed the emergence of resistance while selection against imatinib (ATP-site inhibitors) allowed growth of resistant clones (Supplementary Fig. 19).

Recent clinical studies of JAK inhibitors suggest that these inhibitors are effective in relieving splenomegaly and constitutional symptoms of myelofibrosis, perhaps by modulating the inflammatory cytokines^18^,^49^. However, unlike imatinib treatment in CML, they have little impact on disease burden. The lack of selective antitumor activity by ruxolitinib and other JAK inhibitors is probably a reflection of both the pathogenetic contribution and dependence on mutant JAK2, thus suggesting that the resistance mutations in patients treated with JAK inhibitors can hardly be detected. We also sequenced JAK2 kinase from four PV patients but could not detect any mutation (data not shown). However, resistance to JAK2 inhibitors will likely emerge when a treatment selective to leukemic clones is used^50^. In proof of concept studies, we demonstrated that resistance was conferred by L983F, but not by Y931C at the maximum tolerated dose, 100 mg/kg. This is probably due to the IC50 of JAK2-V617F/Y931C (~1.0 μM), which is 4 fold lower than the C_max_ (4.4 μM) for ruxolitinib at 100 mg/kg^51^. Thus, it appears that many mutations can be suppressed by dose escalation, but the resistant variant L983F will pose significant challenge that can be targeted by either fedratinib or CYT-387.

Drug resistance to kinase inhibitors poses a great challenge for effective therapeutic response. One strategy to combat resistance is to design new ATP-site inhibitors that derive selectivity by alternative modes of binding, such as with BCR-ABL inhibitors dasatinib^52^, AP24163^53^, AP24534^54^ and DCC-2036^55^. An alternative strategy is to target the allosteric sites by small molecule inhibitors as with the BCR-ABL inhibitors GNF2 and GNF5^56^. Allosteric inhibitors have shown greater target selectivity and suppression of resistant variants but tend to select a different constellation of mutations because of differences in their binding sites. Therefore, strategies using combinations of allosteric and ATP-site inhibitors were pursued that, indeed, demonstrated effectiveness in suppressing the emergence of resistant variants^56^. Although such combinations were effective, they preferentially select resistant clones with multiple mutations, presenting a formidable challenge for therapeutic targeting^53^,^56^. In this study we demonstrate that targeting the substrate-binding pocket is more effective in combating genetic resistance because mutations in this region may either block the substrate-binding/phosphorylation or produce a catalytically defective enzyme, which will be counterselective. Additionally, targeting substrate-binding-site may provide better selectivity than ATP or allosteric site inhibitors due to the rich variations in substrate-binding pocket to accommodate differential substrate specificity. This study presents a paradigm that should be extended to other oncogenic kinases for developing inhibitors targeting the peptide substrate-binding pocket to combat the emergence of genetic resistance.

In conclusion, we demonstrate that like other kinases JAK2 is prone to develop resistant mutations against ATP site inhibitors, but targeting the substrate-binding site prevents the selection of resistant mutations. This study provides a proof-of-concept for the therapeutic targeting of substrate-binding site of oncogenic kinases and establishes the rationale for clinical evaluation of this concept.

## Methods

### Ethics statement

The study involving mice conformed to the National Institutes of Health guide for the care and use of laboratory animals and was approved by the Cincinnati Children’s Hospital Institutional Animal Care and Use Committee (IACUC). The study involving human participants was approved by the institutional review board (IRB) of Cincinnati Children’s hospital. The methods were carried out in accordance with the approved guidelines. All clinical research was performed on the basis of the principles expressed in the declaration of Helsinki.

### Reagents

Plasmid constructs pMSCV-JAK2V617F-puro and pMSCV-JAK2-puro were provided by Dr. Ross Levine, and pMSCV-TEL-JAK2-Ires-GFP, pMSCV-TEL-JAK1-Ires-GFP, pMSCV-TEL-JAK3-Ires^57^-GFP, pMSCV-STAT5-Ires-GFP, pMSCV-STAT5-1*6-Ires-GFP, and pMSCV-BCL2-Ires-GFP were provided by Dr. Garry Gilliland. JAK2 and ABL inhibitors were purchased from Chemietech Inc. and LC Chemicals.

### JAK2 mutagenesis and screening

To generate random mutation library of Jak2-V617F, E. coli XL1-red cells were transformed with one μg of JAK2-V617F plasmid DNA. More specifically, 50–100 ng of DNA (more than 100 ng of DNA decreases transformation efficiency) was mixed with 100 μl of competent cells in a pre-chilled polypropylene tube and incubated on ice for 30 min, gently swirling every 5 min. For a good library, four to six tubes of competent cells are used. Cells from each tube were plated on four 10 cm LB-agar plates containing 100-μg/ml ampicillin. Plates were incubated at 37 °C for 18–24 hours. When colonies became visible (18–24 hrs), cells were pooled from each plates and plasmid DNA was isolated using the Qiagen’s midiprep kit. At this stage, the heterogeneity of mutations in the library can be roughly assessed by restriction digestion with a frequent cutter such as Sau3A1 or Taq1 or sequencing.

To select resistant clones of JAK2-V617F, high titer retroviruses were produced as describe^58^. One hundred million native BAF3 cells were transduced with 100 ml of virus supernatants having a viral titer of 0.5–1 × 10^6^. We did not used BaF3 cells expressing homodimeric receptor such as EPOR and Mpl, which has been used in other studies for screening. We observed that the overexpression of EPOR or MPL tend to loose dependence on Jak2 resulting to emergence of false-positives^58^. More specifically, 100 × 10^6^ cells were mixed with 100 ml of virus supernatant and 100 ml of RPMI, containing 10% serum and 24 μg/ml of polyberene. These cell mixers centrifuged at 2500 rpm for 90 minutes at 37 °C. After centrifugation, the plates were transferred to incubator for 24 hour incubation at 37 °C. Next day, cells were pooled together and mixed with ruxolitinib and medium containing soft-agar plated in six well plates Plates were incubated at 37 °C for two weeks. Resistant colonies were picked and sub-cultured individually in 24 well plates for 3–4 days.

#### Sequencing and alignment

Expanded colonies were harvested 7 to 14 days after isolation from agar, whole genomic DNA extracted using the DNeasy genomic (Qiagen, USA), and whole (3.5 kB) JAK2 was amplified by PCR using forward and reverse primers (5′- ATGGGAATGGCCTGCCTTACAATG-3′ and 5′- TCACGCAGCTATACTGTCCCGG-3′, respectively) and long template high-fidelity polymerase (Roche Applied Science, USA). Coding region was next sequenced by 8 different primers spanning the full coding region: P1, 5′-CGACGGCCAGTCTTAAGCTCG-3′; P2, 5′-GTAGTGGCAGCAGCAGAACC-3′; P3, 5′-GGAATTCAGTGGTCAAG-3′; P4, 5′-GAGAATACAACCTCAGCG-3′; P5, 5′-GATCACTGGATACATACC-3′; P6, 5′-GCATGGACTATGAGCCAG-3′; P7, 5′-CATATGGAAGTTTACGAG-3′; and P8, 5′-TGGAGTCTGGTCTTTCTCC-3′. Sequence alignment was performed using the Lasergene Genomics Suite (DNASTAR).

#### Generation of mutants

Mutants isolated in the screen were engineered into pMSCV-JAK2-V617F-Puro, using QuikChange XL mutagenesis kit (Stratagene). Each point mutants were confirmed by sequence analysis.

### Cell-viability, kinase-inhibition and western-blotting assays

Ba/F3 cells expressing mutant BCR-ABL proteins were plated in 96-well plates (0.5–1 × 10^4^ cell per well) in RPMI medium containing 10% FCS and lacking IL-3. Kinase inhibitors were included in the media at varying concentrations. After 60 hours, the number of viable cells was assessed using WST-1 reagent (Roche Applied Science, USA), according to the manufacturer’s specifications. Assays were performed in quadruplicate. Absorbance measurements at 450 nm were averaged and plotted against inhibitor concentration as a best-fit sigmoidal curve, using the Origin 7.0 non-linear curve-fitting algorithm (Origin Lab, USA); the concentration resulting in 50% maximal inhibition was reported as the cellular IC50. BCR-ABL kinase assay and immunoblotting were performed as previously described^28^,^32^.

### Structural modeling, inhibitor docking and representation

Structural models of JAK2 domains were built by homology-based modeling using SWISS-MODEL^27^ and crystal structures. SH2 and pseudokinase domains were built using the crystal structures of JAK2 kinase domain (PDB: 2B7A and 3UGC), ABL kinases (PDB: 1OPJ, 1OPL, 2G1T, 2G2F, 2G2I, and 2G2H) and SRC kinase coordinates (PDB: 2SRC and 1OPK); FERM domain was built using the crystal structure of focal adhesion kinase crystallized with FERM domains (PDB: 2J0J and 2J0L).

Targeted docking to the ATP-site and blind docking to whole-kinase domain structure were performed using SwissDock^35^,^59^ and JAK2 kinase coordinates (PDB: 2B7A and 3UGC). SwissDock is based on EADock DSS docking software^59^. The SwissDock algorithm works by generating a large number of binding modules (typically 5000 to 15,000)—either by local docking (in a user-defined box) or on the entire protein surface (blind docking), which identifies unanticipated binding sites and cavities. Simultaneously, CHARMM energies are estimated on a grid^60^ and ranked—taking into account the solvent effect, using the FACTS implicit solvation model. Modules with the most favorable energies are clustered^61^, and the most favorable clusters are contained in the resulting PDB file. For each cluster, binding modules with the lowest energy (i.e., the most likely to represent true binding) were selected for further verification. Figures were generated using PyMol.

#### Docking simulations

For further validation and comparison, docking simulations were performed using AutoDock version 4^62^. Two alternative JAK2 protein structures available from the Protein Data Bank (3TJD, 2B7A) were used to assess the effects of structural details using rigid protein docking mode in AutoDock. Three different grid boxes were used to further assess specificity of binding: box 1 covering the ATP binding site, box 2 covering the substrate-binding site, and box 3 covering both sites (and effectively the entire protein). For 3TJD structure, these grid boxes were defined by the following centers and side dimensions (in Angs): box1(center: X: 113, Y:64, Z:9; side: X:50, Y:50, Z:50), box2 (center: X: 102, Y: 60, Z:-7; side: X:60, Y:54, Z:58), and box3 (center: X:102, Y:6, Z:2; side: X:126, Y:126, Z:126). The corresponding boxes for 2B7A were defined as follows: box1 (center X:114, Y:64, Z:7; side: X:46,Y:46,Z:46), box2 (center X:100, Y:60, Z:-4; side: X:50, Y:50, Z:60), and box3 (center: X:107, Y:62, Z: 1.5; side: X:126, Y:126, Z:126). In the case of ATP binding site grid box was centered around residues: 1013, 1015, 1019, 1022, 1029, and 1031. ZINC19862646 (SAR302503 (fedratinib)) and ZINC43207851 (Ruxolitinib) were used as ligands. The following parameters were used for docking simulations: ga_pop_size 150, ga_num_evals 50000000, and ga_run 200, with default spacing for grid boxes. The results were analyzed using Polyview-MM^63^.

#### MD simulations

With the goal of further validating docking predicted poses and assessing the role of conformational flexibility, all-atom molecular dynamics (MD) simulations of receptor-ligand complexes were performed using the AMBER package^64^. Standard protocols for MD simulations were used as follows. AMBERTOOL14^65^,^66^ and general AMBER force field (GAFF)^67^ were used to create parameter and coordinate files for the ligand. Hydrogen atoms were added to the ligand with the help of Chimera^68^. AMBER force field ‘ff12SB’^69^ was used for the protein and TIP3P waters for explicit solvent model^70^, using periodic boundary conditions with 17277 water molecules in a truncated octahedron box so that all atoms were no less than 15 Å from the side of the box. The protein-ligand system was subjected to minimization, equilibration and all-atom MD simulation during which (a) hydrogen atoms were kept fixed using SHAKE algorithm^71^, (b) periodic boundary condition with a non-bonded cutoff of 10 Å was applied, (c) long range electrostatics effects were accounted for using PME^72^.

A minimization of 10,000 steps were done applying a harmonic potential of force constant 100 kcal/mol Å^2^ to constrain the motion of atoms of the protein-ligand complex but allowing to move water molecules freely. For the temperature equilibration, the temperature was raised gradually from 0 K to 300 K in three phases: (i) from 0 K to 50 K, (ii) from 50 K to 150 K and (iii) from 150 K to 300 K at constant volume. There were 200000 steps of a 200 ps MD simulation with time step of 0.001 ps. A force constant of the harmonic potential was 10 kcal/mol Å^2^ on solute atoms. For the equilibration of the solvent density at constant temperature 300 K and pressure of 1 atm, a 200 ps MD simulation with Langevin dynamics of collision frequency 1 ps^−1^ was run in 200000 steps. Finally, in the production runs 50 ns MD simulations at constant temperature of 300 K and a constant volume were performed for each of the two binding sites and the corresponding complex with the ligand either in ATP or substrate-binding pocket. For each of the sites, 50,000 snapshots of the complex were obtained and analyzed with AMBERTOOL14, xmgrace and awk. In the simulation, sander and PMEMD were used with MPI run and CUDA as needed in GPU environment.

### Protein expression and purification

Proteins were expressed in *Spodoptera frugiperda,* Sf9 cells. Full-length Jak2-V617F and kinase domain having Strep-tags (WSHPQFEK) at c-terminus were cloned in pENTR222 (Invitrogen) using polymerase chain reaction (PCR). Point mutations in to the full-length Jak2-V617F (R277K, G417P, D544H, L551P, R683T, Y931C, L983F) and kinase domains (E930G, Y931C and L983F) were created by site directed mutagenesis using QuikChange XL mutagenesis kit. Each point mutants was confirmed by sequence analysis. These pENTR-JAK2-V61F and pENTR-JAK2-JH1 constructs were used to develop baculoviruses using Baculodirect kit (Invitrogen), according to manufacturer’s instructions. A high titer virus (10^9^–10^10^ pfu/ml) was generated for each construct for protein expression. Three hundred mls of suspension culture of insect cells at a density of 2–3 × 10^6^/ml were infected with viruses at 5-10 MOI (multiplicity of infection). Infection media was supplemented with 10% FBS as in the absence of serum expression is extremely low and often beyond detection level by western blotting. After 60 hrs of infection, cells were harvested for protein purification. All purification steps were carried out at 4 °C. The cells were lysed in lysis buffer (50 mM potassium phosphate buffer pH 8.0, 300 mM NaCl, 1% Triton X-100, 5% glycerol, 1 mM EDTA, 1x complete protease inhibitors cocktail (Roche Diagnostics). Cell lysates were incubated with Strep-Tactin resin (IBA) for two hrs at 4C, followed with batch purification. The JAK2 proteins were eluted in elution buffer (50 mM potassium phosphate buffer pH 8.0, 300 mM NaCl, 5% glycerol and 2.5 mM desthiobiotin). Elution buffer was exchanged with storage buffer (50 mM potassium phosphate buffer pH 8.0, 300 mM NaCl, and 6% glycerol) by Amicon ultrafilters.

Protein concentration and purity was determined by Bradford assay and SDS-PAGE analysis, respectively.

### Kinase Assays and steady state enzyme kinetics

Kinase assays were performed in triplicate, using the ADP-Glo kinase-assay kit (Promega, USA), recombinant JAK2 purified from insect cells (Signalchem Inc.), and STAT5-derived peptide (AKAADGYVKPQIKQVV) as a substrate (synthesized by Genscript Inc). These assays were performed in triplicate in 96-well plates. For the ATP competitive assay, for each reaction, 50 ng of recombinant JAK2 was incubated with a graded concentration of inhibitor and 50 μM STAT5 peptide at different ATP concentrations (10, 100, 500, and 1000 μM). For the substrate competitive assay, 50 ng JAK2 was incubated with 50 μM ATP and a graded concentration of inhibitor at different concentrations of STAT5 peptide (50, 250, 500 and 1000 μM). Reaction mixes were incubated at 37 °C for 30 minutes. Next, an equal volume (25 μl) of ADP-Glo was added and plates were incubated at room temperature for 30 minutes, to deplete the remaining ATP and terminate the kinase reaction. Next, 50 μl of kinase detection reagent was added to each well to convert ADP to ATP that was measured by luciferase/luciferin reaction. Luminescence was measured 1 hour after adding the Kinase Detection Reagent during this time plates were incubated at room temperature. Luminescence correlates with the amount of ADP (i.e., kinase activity), and was recorded with GloMax 96 Microplate luminometer (Promega, USA) and plotted with Prism software (Graphpad).

### Labelfree MicroScale Thermophoresis (MST) binding assay

Labelfree MicroScale Thermophoresis binding experiments were carried out with 40 or 1500 nM target protein in 25 mM Tris-HCl pH 7.5 + 150 mM NaCl + 0,1% Tween with varied concentrations of small compound at 40% MST power, 80% LED power in standard zero background capillaries on a Monolith NT.labelfree device at 25 °C (NanoTemper Technologies, Munich, Germany). For fedratinib a ligand dependent quenching effect was detected. Due to this, the raw fluorescence was used for analysis. For the compound ruxolitinib the Thermoporesis + TJump was analyzed. The recorded fluorescence was normalized to fraction bound (0 = unbound, 1 = bound), processed using the software KaleidaGraph 4.5 and fitted using the Kd fit formula derived from the law of mass action. Technical duplicates were performed for each experimental setup.

### Fluorescent MicroScale Thermophoresis (MST) binding assay

Fluorescence MicroScale Thermophoresis binding experiments were carried out with 5 nM Cy5-labeled target protein in 25 mM Tris-HCl pH 7.5 + 150 mM NaCl + 0,1% Tween with varied concentrations of small compound at 40% MST power, 20% LED power in hydrophilic capillaries on a Monolith NT.115 pico device at 25 °C (NanoTemper Technologies, Munich, Germany). For fedratinib a ligand dependent quenching effect was detected. Due to this, the raw fluorescence was used for analysis. For the compound ruxolitinib the Thermoporesis + TJump was analyzed. The recorded fluorescence was normalized to fraction bound (0 = unbound, 1 = bound), processed using the software KaleidaGraph 4.5 and fitted using the Kd fit formula derived from the law of mass action. Technical duplicates were performed for each experimental setup.

### *In vivo* efficacy and resistance study of JAK2-V617F variants

BaF3 cells expressing native JAK2-V617F and resistant variants Y931C and L983F were transduced with retrovirus co-expressing firefly luciferase and Cherry fluorescent protein. HEK293T cells were transfected with pMSCV-Luc-cherry-GW (firefly luciferase amplified from pLVX-luciferase by PCR and cloned in pMSCV-cherry-GW vector) to generate high titer viruses. For engraftment, 1 million cherry-positive cells were injected into tail veins of syngeneic female Balb/c mice (Jackson Laboratories, USA). Starting 3 days later, mice were IP injected twice daily with 100 mg/kg of ruxolitinib. On days 7 and 14, mice were IP injected with luciferin (Promega) and imaged on an IVIS Spectrum imager (Caliper LifeSciences, USA); images were analyzed using Living Image software. All experiments involving mice conformed to the National Institutes of Health Guide for the care and use of laboratory animals and were approved by the Cincinnati Children’s Hospital Institutional Animal Care and Use Committee (IACUC).

### Sequencing of Patient samples

Bone marrow mononuclear cells from PV patients were collected with informed consent. Institutional Review Board (IRB) approval was obtained from Cincinnati Children’s hospital or UCSF. The JAK2 cDNA was amplified using PCR. PCR products were sequenced and analyzed using the Lasergene Genomics Suite (DNASTAR) to identify any mutation.

## Additional Information

**How to cite this article**: Kesarwani, M. *et al.* Targeting substrate-site in Jak2 kinase prevents emergence of genetic resistance. *Sci. Rep.* **5**, 14538; doi: 10.1038/srep14538 (2015).

## Supplementary Material

Supplementary Information
