# Supplementary material for: Targeting substrate-site in Jak2 kinase prevents emergence of genetic resistance
Source: Sci Rep. 2015 Sep 30;5:14538. doi: 10.1038/srep14538 (PMC4588578; doi:10.1038/srep14538)
Supplement: Supplementary Information [file srep14538-s1.pdf]

Supplementary Information

Targeting substrate-site in Jak2 kinase prevents emergence of genetic resistance

Meenu Kesarwani[1], Erika Huber[1], Zachary Kincaid[1], Chris R Evelyn[1], Jacek Biesiada[1], Mark Rance[2], Mahendra B. Thapa[2] Neil P Shah[3], Jarek Meller[1], Yi Zheng[1] and Mohammad Azam[1,2 4]

Supplementary Table 1: IC_{50} values of resistant varinats against other JAK2 inhibitors. IC_{50} measured in transformed BAF3 cells with and without IL3. Structural domains are indicated on the left side of table.

		IC50 [nM]									
		AZD1480		CYT-387		INCB018424		Lestaurtinib		TG101348	
		- IL3	+ IL3	- IL3	+ IL3	- IL3	+ IL3	- IL3	+ IL3	- IL3	+ IL3
FERM	Y44C	448	597	1282	1438	397	618	138	108	974	978
	P58A	410	470	988	2651	194	760	108	113	984	991
	H110R	458	494	850	1462	162	755	98	107	589	978
	M181R	162	672	698	1165	112	1052	73	125	506	993
	N99S	391	995	906	1927	166	434	138	105	681	993
	R277K	417	854	1214	2190	186	587	153	111	937	983
	V366G	393	720	930	2021	135	416	95	107	703	974
SH2	G417P	415	730	1009	1750	195	442	108	103	977	994
	M483R	203	630	826	1380	136	461	80	107	533	938
Pseudokinase	L551P	250	888	855	1303	172	482	95	111	924	961
	TF556PS	151	730	650	1130	112	416	47	108	496	906
	V582A	437	706	1010	3066	395	431	407	105	1000	953
	E596Q	255	1054	992	2315	414	760	100	433	927	1016
	G652E	437	806	1009	860	424	672	370	165	1015	1012
	L680P	195	831	792	886	127	433	88	125	569	962
	R683T	164	695	470	2099	128	515	78	119	980	841
	E684Q	409	676	490	481	385	490	92	115	735	965
	N731Y	374	831	938	2075	207	576	101	121	955	990
	D812G	415	663	993	2190	371	426	105	114	932	944
	P823L	443	674	976	2998	407	488	100	110	940	1000
Linker	D840N	406	698	946	4050	370	1022	100	115	906	972
KINASE	Q853P	160	946	713	2341	203	1076	67	120	592	983
	L902M	471	935	995	2190	422	1157	151	122	922	960
	N924T	229	690	813	1144	401	420	100	113	930	970
	L927F	145	730	688	1170	99	490	58	180	517	983
	Y931C	2388	9000	1012	3314	562	1008	99	431	948	1400
	Y931F	432	412	714	1192	115	402	92	132	572	970
	Y966E	220	458	776	1033	108	280	90	108	522	970
	R971G	223	768	833	1120	336	3742	92	133	692	982
	D976N	144	738	696	110	98	200	42	122	536	986
	N981S	99	795	491	1125	70	473	40.6	109	442	960
	L983F	1136	2771	971	1042	1331	10980	427	170	953	978
	N986Y	219	648	713	1055	122	474	85	114	609	973
	E987D	394	958	991	3858	185	671	58	130	925	1009
	L1001W	178	702	795	1061	115	500	49	118	584	967
	E1006K	250	1061	906	1050	136	918	71	114	1243	970
	E1046K	186	909	828	1103	106	495	86	155	603	980
	R1063P	134	754	744	3840	108	565	92	188	548	988
	E1080K	337	693	998	1174	186	450	116	160	963	1005
JAK2-V617F		152	268	704	1042	100	210	89	110	610	1070

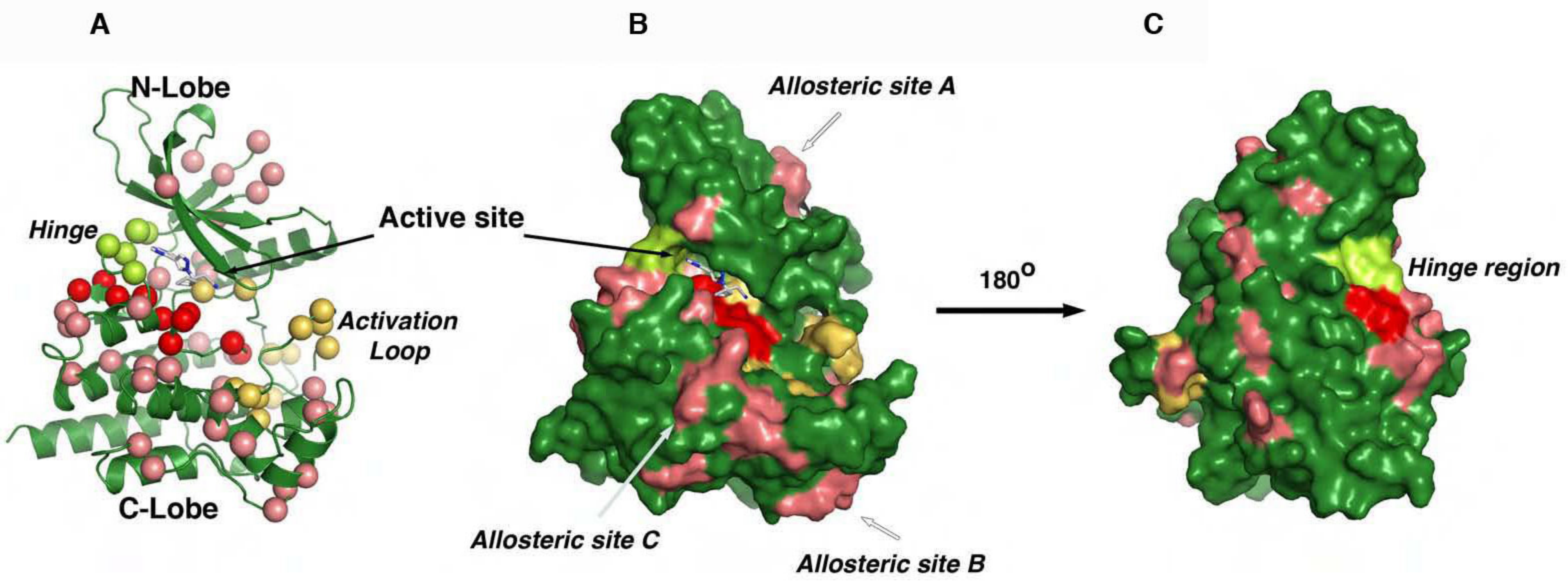

Supplementary Figure 1:
Mapping of resistant mutations on the surface of JAK2 kinase identified four allosteric sites.
A. A ribbon depiction of JAK2 kinase showing the ruxolitinib resistant mutations (colored circles).
B, and C, surface depiction of JAK2 kinase domain identified mutational hot-spots as cluster.
Mutations clustered in active site are marked with black arrow while allosteric sites A, B and C marked by filled and gray arrows

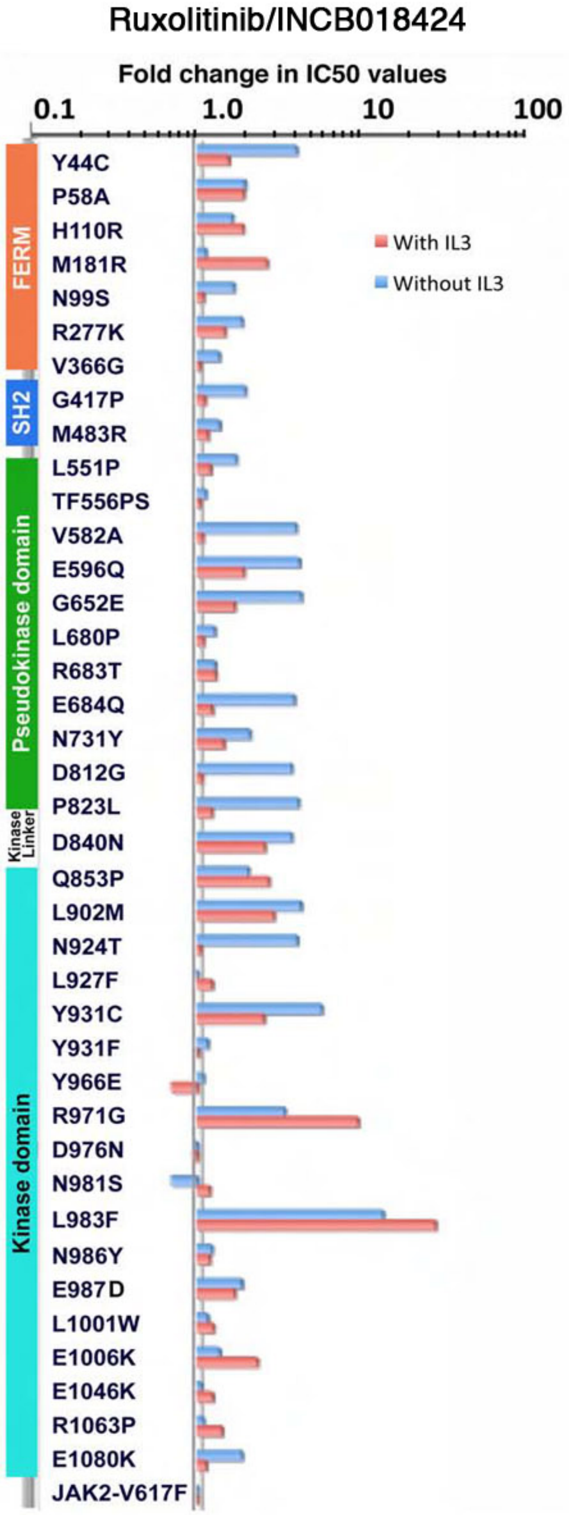

Supplementary Figure 2: JAK2-V617F variants confer resistance to Ruxolitinib. Bar graph showing the fold difference in the IC50 values, relative to native JAK2-V617F.Values were normalized to 1 against JAK2-V617F and plotted on a semilogarithmic scale. Structural domains, FERM, SH2, pseudokinase and kinase are indicated on the left side.

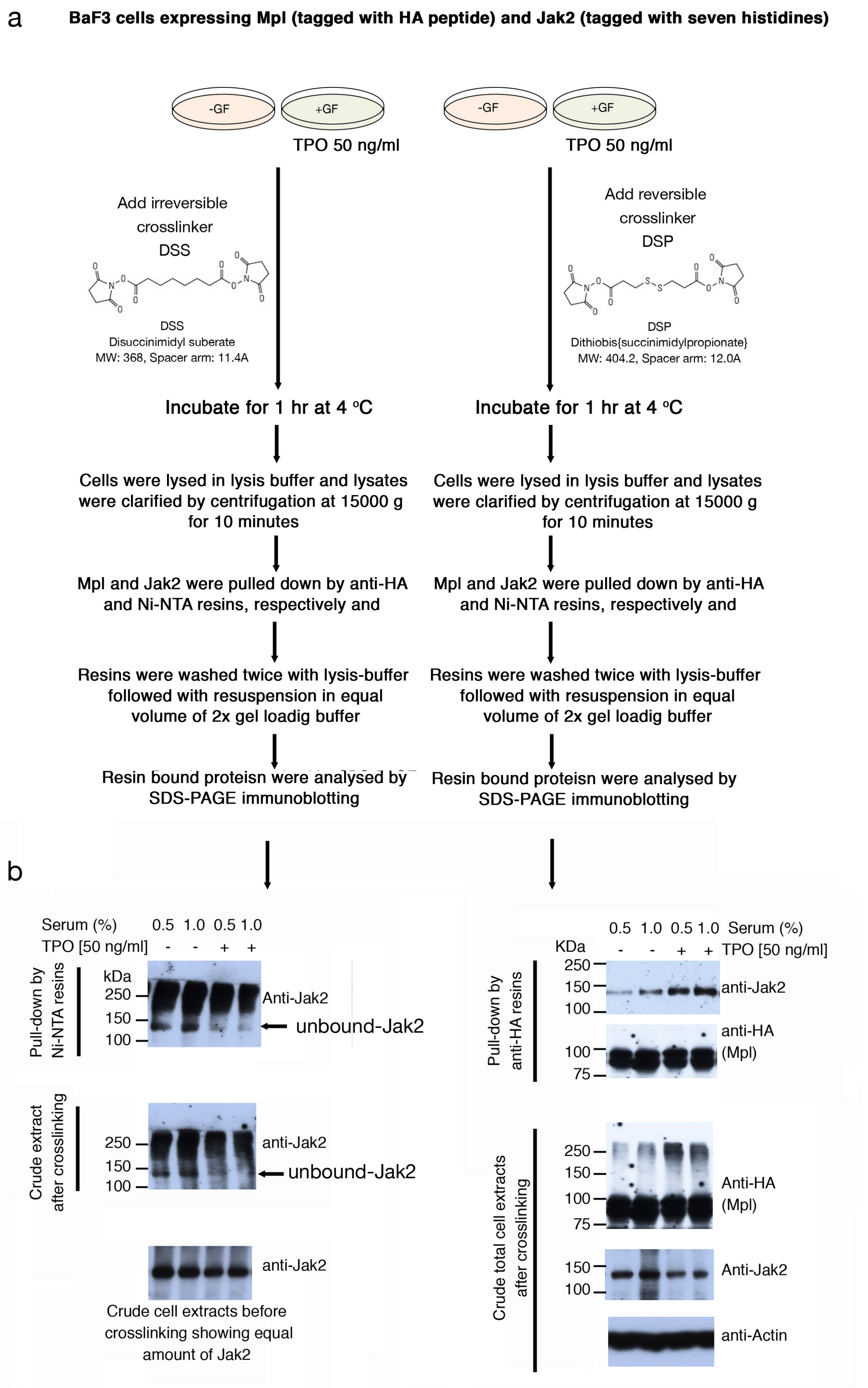

Supplementary Figure 3: Receptor activation by cytokines induces complete binding of Jak2

a. Showing the schema for analyzing the receptor-bound and free form of Jak2.

b. Immunoblots probed with anti-Jak2 from the extracts crosslinked with irreversible linker (DSS) showing complete translocation of Jak2 to higher molecular weight complexes when activated by TPO (left panel). Note, a receptor free, perhaps autoinhbited form of Jak2 are more prominent in the absence of TPO, (marked by arrow in the left panel).Thus providing an experimental support for the autoinhibited Jak2 model proposed by Jim Ihle and Lily Huang's group (Funakoshi-Tago et al. 2008,Mol. Cell. Biol. 28, 1792–1801and Zhao L et. al. Biochem. J. 2010, 426, 91–98)

Right panel, showing the interaction of Mpl and Jak2 in the presence and absence of TPO (Mpl ligand) Please note, more binding of Jak2 with Mpl in the presence of TPO (left panel) and

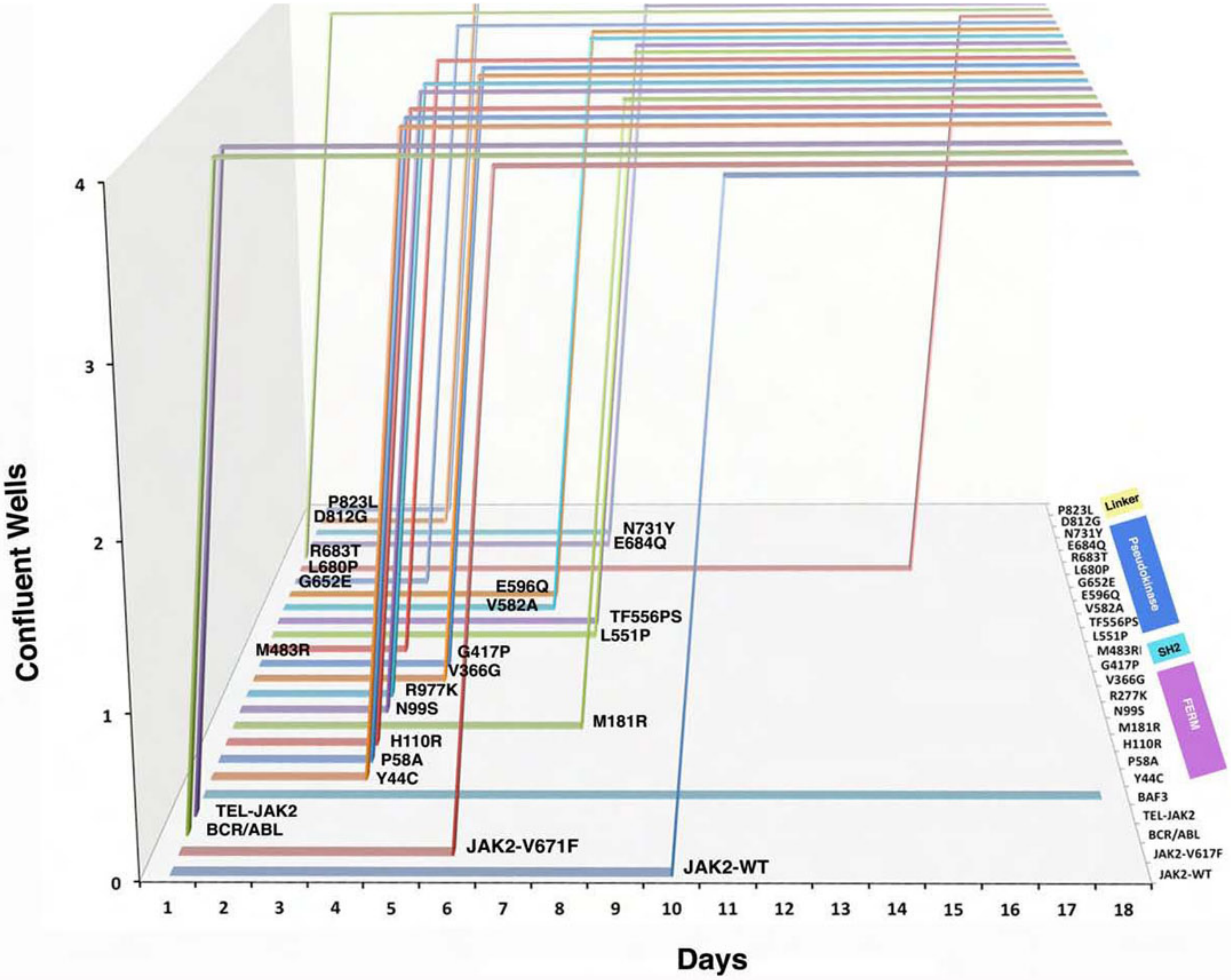

Supplementary Figure 4 A: Ruxolitinib resistant mutations have altered transformation potential. Cell proliferation assay of BaF3 cells expressing BCR-ABL, TEL-JAK2, JAK2-WT, JAK2-V617F and JAK2-V617F-variants. Cells were plated in quadruplicate in 96- well plates at a density of 5,000 cells per well in the absence of IL-3, and scored when the wells became confluent.

A. Cell proliferation assay of resistant variants from FERM, SH2 and pseudokinase domain (shown on the right). Note that, like BCR/ABL and TEL-JAK2, the variant R683T has acquired full transformation potential.

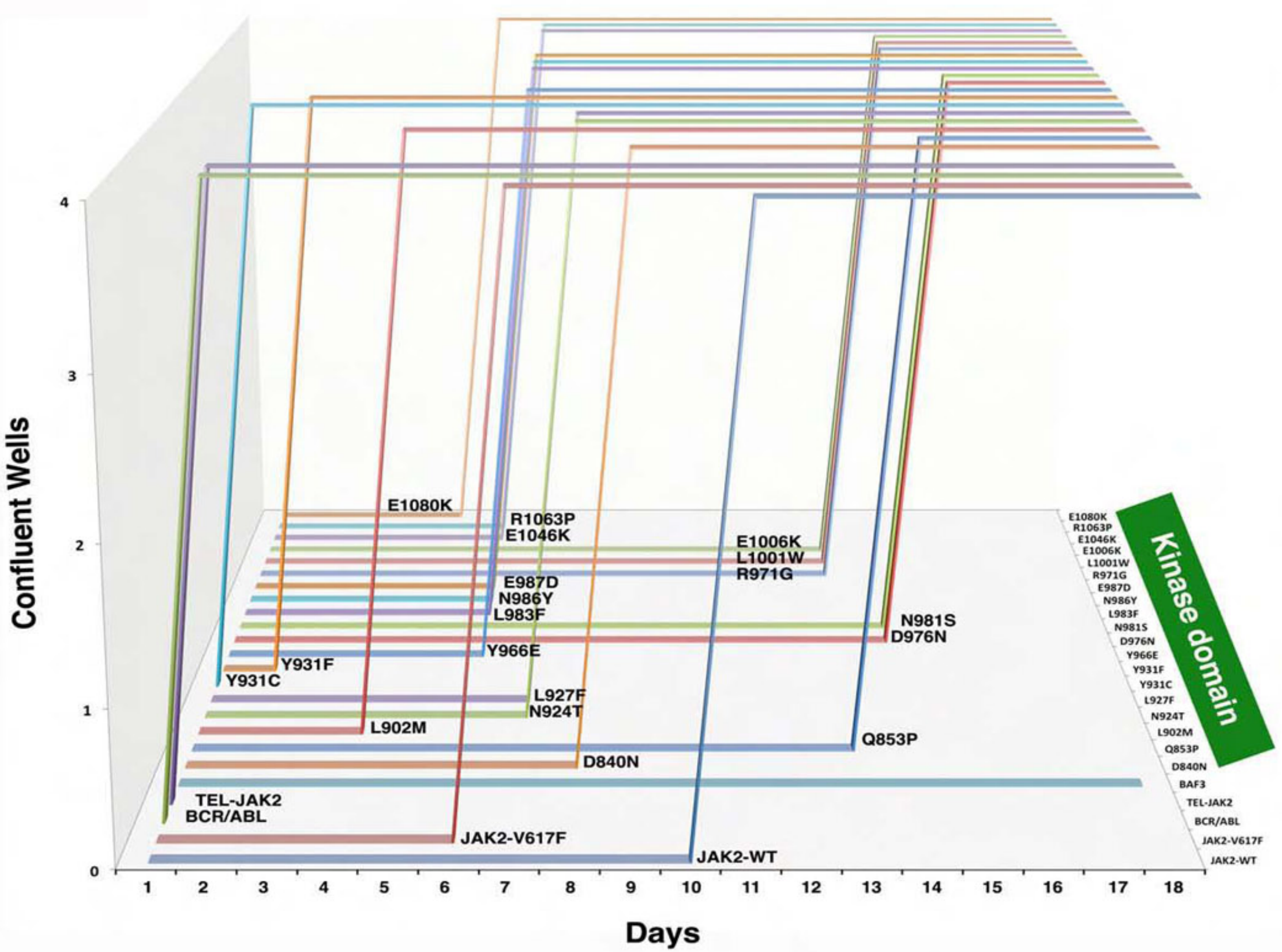

Supplementary Figure 4 B: Ruxolitinib resistant mutations have altered transformation potential. Cell proliferation assay of BaF3 cells expressing BCR-ABL, TEL-JAK2, JAK2-WT, JAK2-V617F and JAK2-V617F-variants. Cells were plated in quadruplicate in 96- well plates at a density of 5,000 cells per well in the absence of IL-3, and scored when the wells became confluent.

B. Cell proliferation assay of resistant variants from the kinase domain. Note that, like BCRABL and TEL-JAK2, variant Y931C/F has acquired full transformation potential, while variants Q853P (P-loop), D976N, N981T (catalytic loop), R971G (helix-E) and L1001W and E1006K (activation loop) have significantly reduced transformation potential (reduced kinase activity).

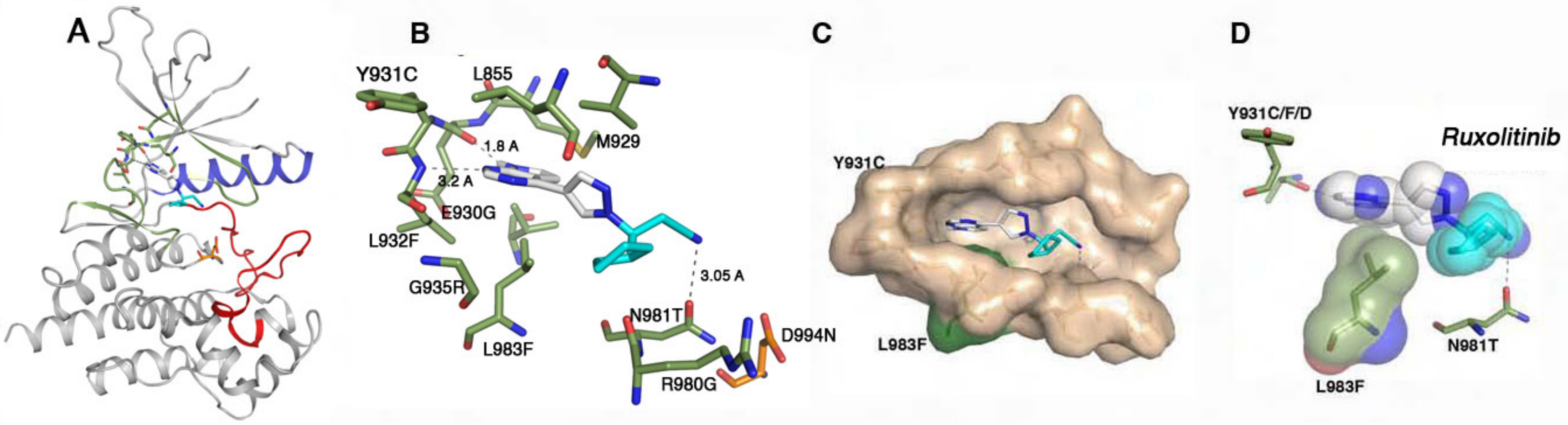

Supplementary Figure 5: Ruxolitinib binds to ATP-binding site in a DFG-in conformation.

A. A ribbon depiction of JAK2 kinase showing Ruxolitinib binds to the active site.

B. Ruxolitinib interacts with seventeen amino acid residues in the active site making three hydrogen bonds with residues Glu 930, Leu 932 and Asn 981 (highlighted as dashed line).

C. A van der Walls surface depiction of the active site residues mediating the interactions with Ruxolitinib.

D. A van der Waals surface depiction of the residue Leu 983 (most frequently mutated residue) showing hydrophobic interaction with Ruxolitinib.

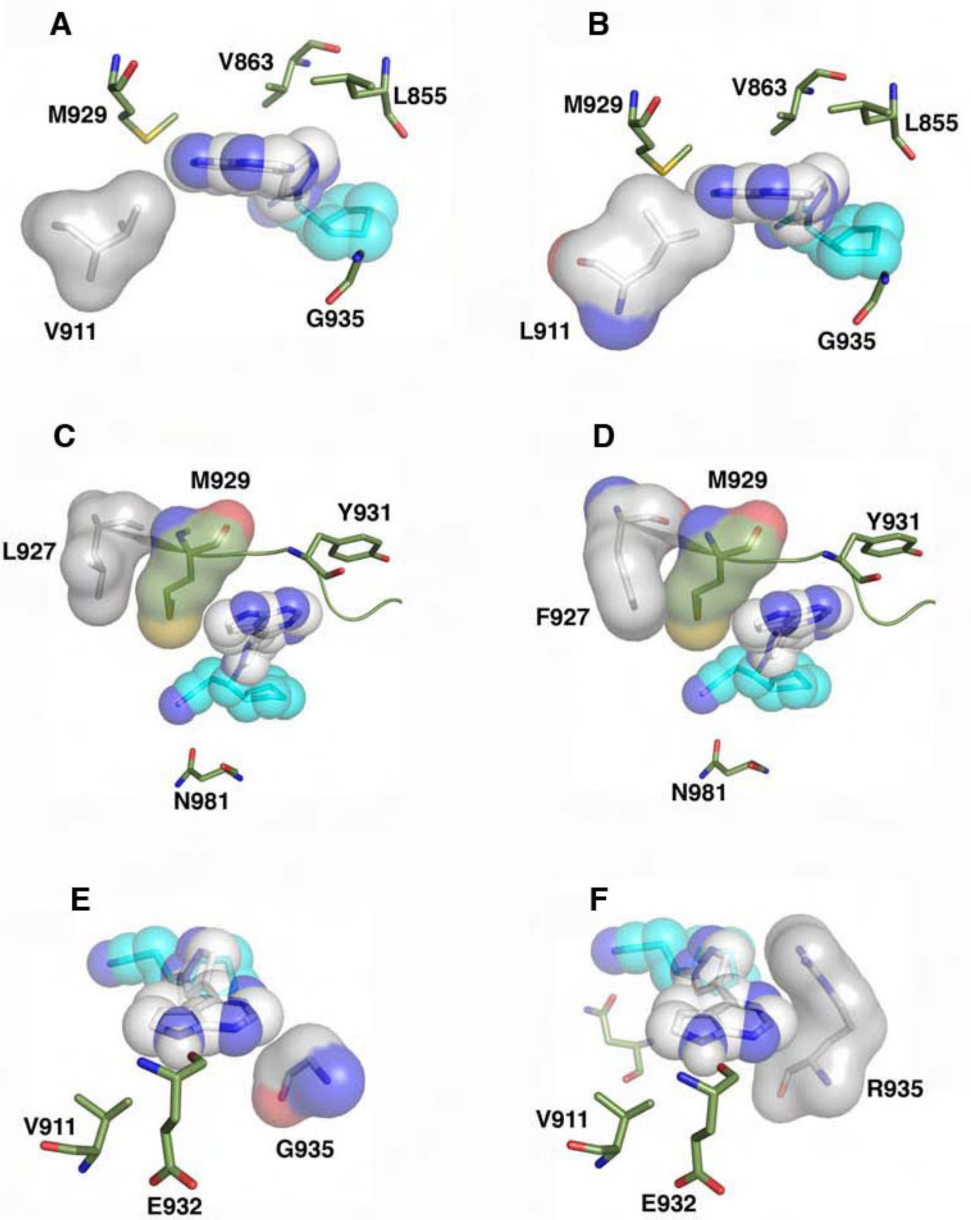

Supplementary Figure 6: Active site residues confer resistance by steric blockade
A and B, substitution of leucine for Val 911 causes direct steric hindrance to Ruxolitinib.
C and D,substitution of phenylalanine for Leu 927 affect drug binding by pushing the Met 929 towards the drug-binding region.
E and F, An arginine substitution for Gly 935 will cause direct steric clash with Ruxolitinib.

.

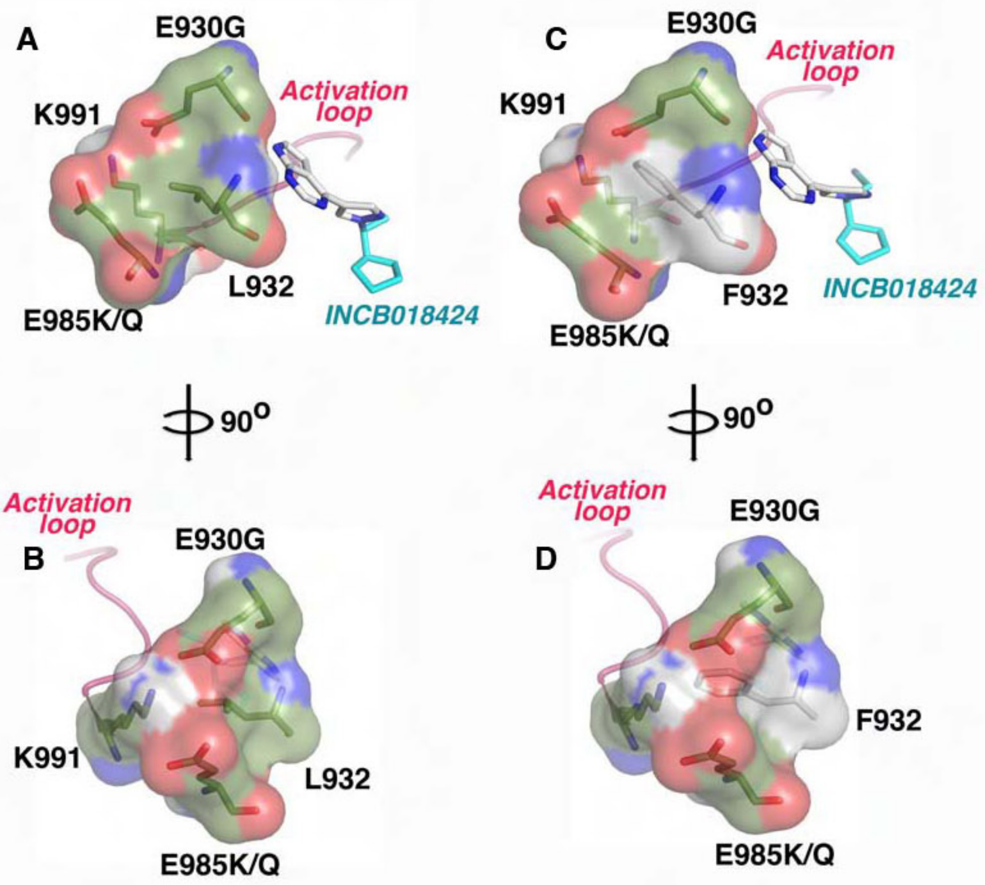

Supplementary Figure 7: Mutations from hinge region destabilizes the active site and DFG motif. Structural modeling of the residues Glu 930, Leu 932 (hinge region) and Glu 985 (catalytic loop)suggest that they stabilize the active state by electrostatic interaction with Lys 991 (N terminus of DFG motif) while packing Leu 932 in between Glu 930 and Glu 985 (A and B). A phenylalanine substitution for Leu 932 will disrupt the electrostatic interactions between Lys 991, Glu 930 and Glu 985. Likewise, substitution of Glycine for Glu 930 and Lysine for Glu 985 (C and D) will disrupt these interactions.

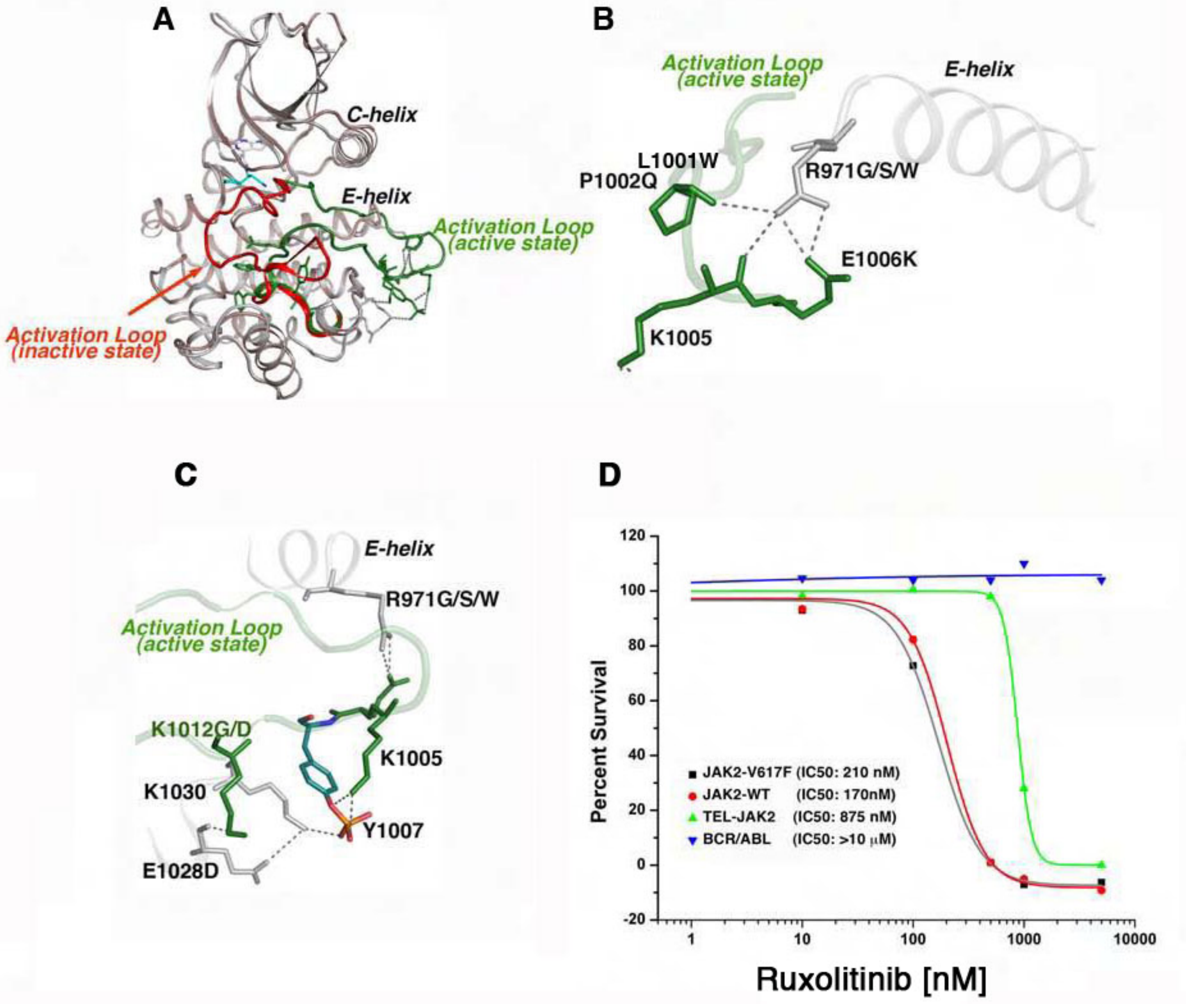

Supplementary Figure 8 : Mutations in Helix-E and activation loop destabilize active state.

A. A cartoon depiction of aligned structures of JAK2 kinase domain in active (PDB: 2B7A) and inactive state (PDB: 3UGC) showing conformation of activation loop in active state (Green) and in inactive state (red).

B. Residue Arg 971 from helix-E stabilizes the activation loop in active state by interacting with Pro 1002, Glu 1006 and Lys 1005 through electrostatic interaction (shown as dashed line).

C. Residues Glu 1028 and Lys 1030 interact with Tyr 1007 when it is phosphorylated suggesting these interactions will stabilize the active state while mutations such as E1028D and K1012G/D will favor for inactive state by destabilizing the active conformation.

D. Dose response analyses for ruxolitinib showing 4-fold higher IC50 value for TEL-JAK2 than JAK2-WT and JAK2-V617F.

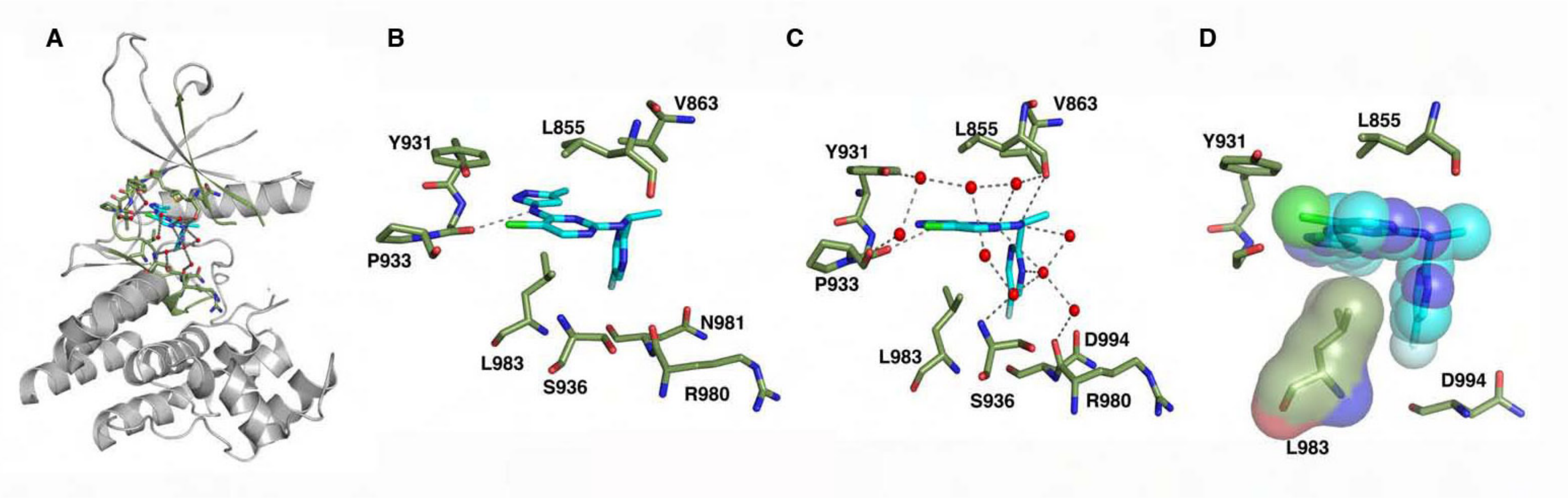

Supplementary Figure 9: AZD1480 binds to the ATP binding site in a DFG-in conformation
A. A ribbon depiction of JAK2 kinase showing the binding of AZD1480 in the active site.
B. AZD1480 anchors to the active site by a hydrogen bond with residue Pro 933 (shown as dashed line) and Van Der Waals interaction with 11 amino acid residues.
C. Tyr 931 stabilizes the active site by hydrogen bond interactions (shown as dashed line) with residues (Leu 855, Ser 836 and Arg 980) mediated by nine water molecules (filled red circle).
A. Surface depiction of the residue Leu 983 (most frequently mutated residue) showing close proximity with AZD1480 and a phenylalanine substitution will confer resistance.

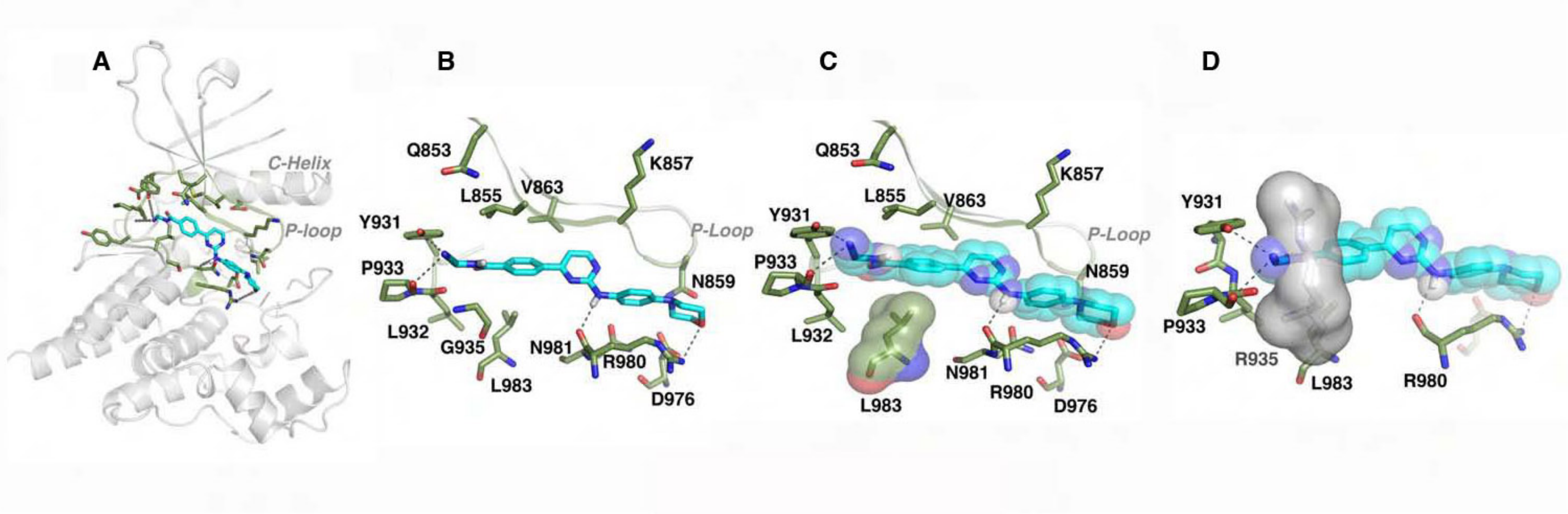

Supplementary Figure 10: CYT-387 binds to ATP-binding site in a DFG-in conformation and Phenylalanine substitution at Leu 983 does not affect its binding.

A. A ribbon depiction of JAK2 kinase showing the binding of CYT-387 in active site.

B. CYT-387 anchors to active site by four hydrogen bonds with the residues Tyr 931, Pro 933, Arg 980 and Asn 981 (highlighted as dashed line) and through van der waals interactions with 13 amino acid residues.

C. A depiction of active site organization and surface depiction of the residue Leu 983 (most frequently mutated residue) showing lack of interaction with inhibitor. Thus, explaining why L983F variant is sensitive to CYT-387.

D. An arginine substitution for Gly 935 will cause steric clash with CYT387.

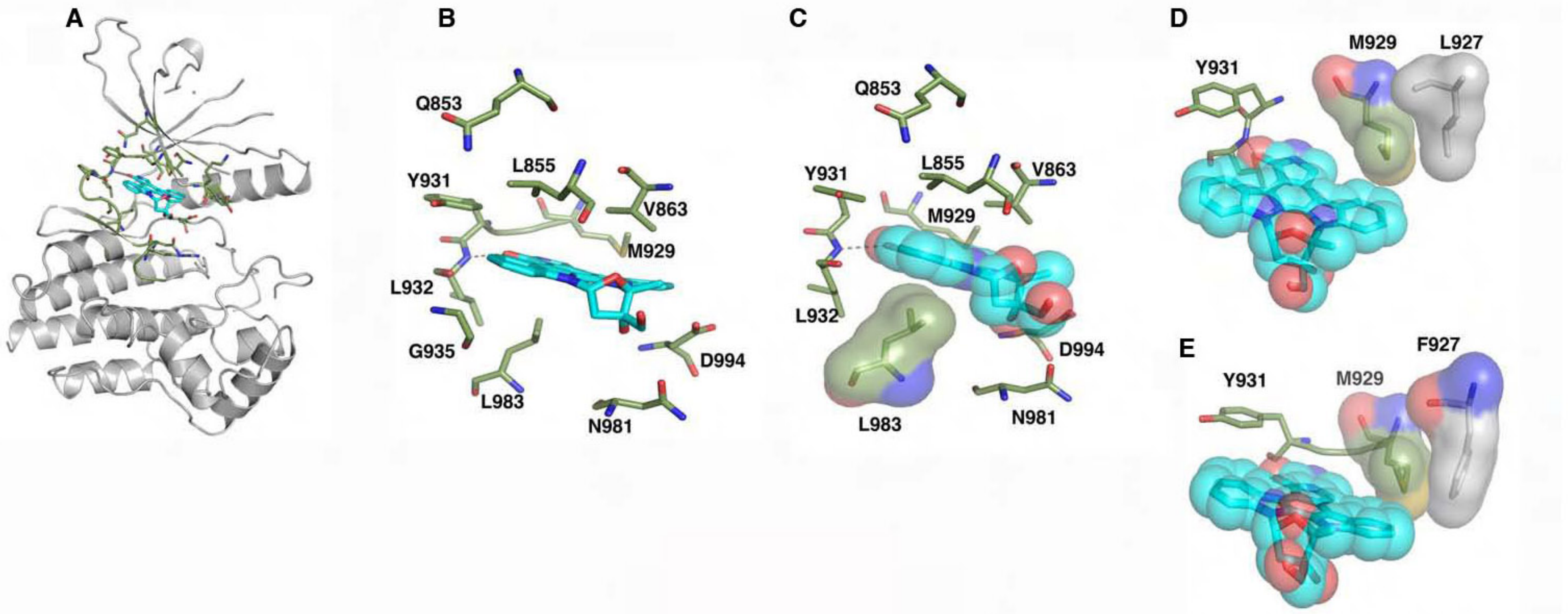

Supplementary Figure 11: Lestaurtinib binds to ATP-binding site in a DFG-in conformation.
A. A ribbon depiction of JAK2 kinase showing binding of lestaurtinib within active site.
B. Lestaurtinib anchors to the active site by a hydrogen bond with rLeu 932 (highlighted as dashed line) and through van der waals interactions with 10 amino acid residues (green sticks).
C. A depiction of active site organization and surface depiction of Leu 983 (most frequently mutated residue) showing close proximity with inhibitor.
D and E. A substitution of phenylalanine for Leu 927 suggests it probably affect drug binding by pushing the Met 929 towards the drug-binding region.

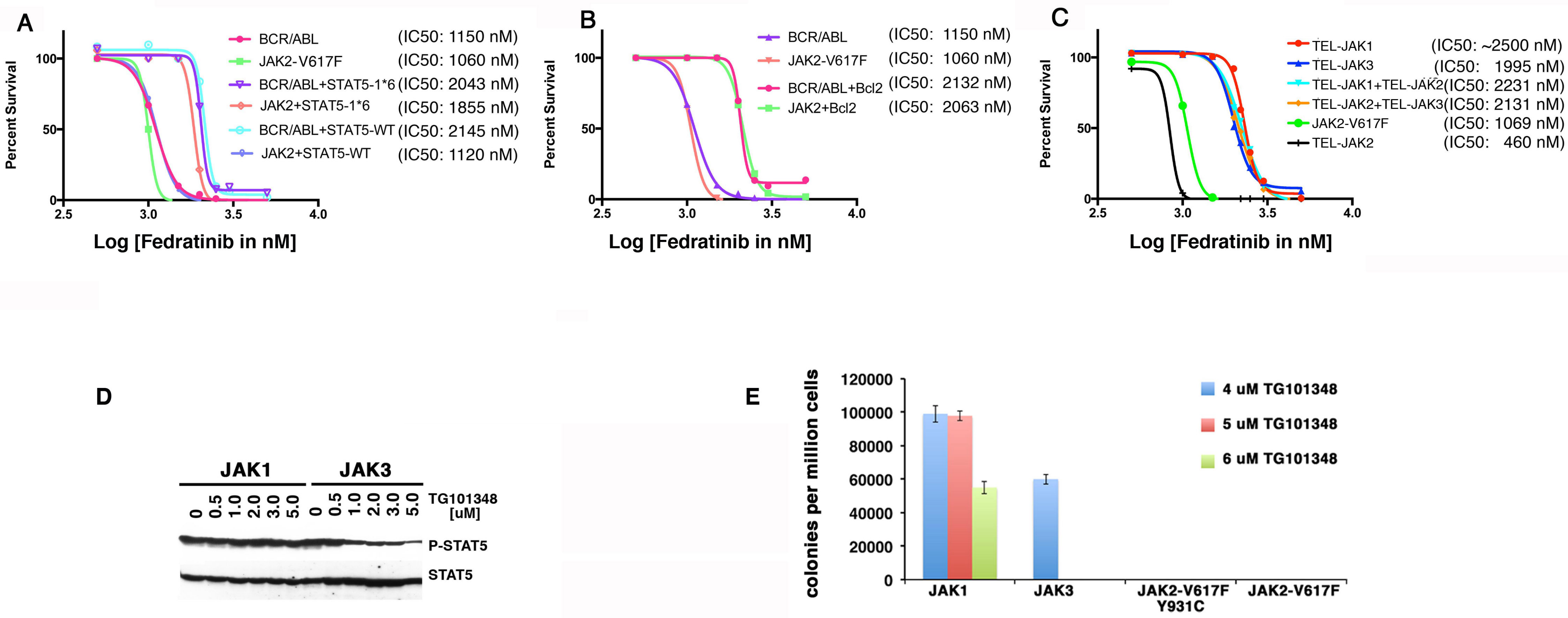

Figure Supplement 12: Overexpression of constitutively activated STAT5, BCL2, JAK1 and JAK3 confer resistance to Fedratinib

A. A dose dependent cell proliferation assay showing increased IC50 for fedratinib by native and constitutively activated STAT5.

B. Likewise, overexpression of BCL2 showed increased IC50 values in cell proliferation assays.

C. Constitutively activated JAK1 and JAK3 (TEL-JAK1 and TEL-JAK3) confer resistance to Fedratinib.

D.. Immunoblot analysis of BaF3 cells expressing TEL-JAK1 and TEL-JAK3 showing phospho STAT5 (upper panel) and total STAT5 (lower panel). Note, dose dependent inhibition of phospho STAT5 in JAK3 while JAK1 is fully resistance up to 5 µM of Fedratinib.

E..Soft-agar colony formation assays showing the emergence of colonies from JAK1 up to 6 µM and JAK3 at 4 µM of Fedratinib. While, JAK2-V617F and JAK2-V617F/Y931C, failed to grow at 4 µM of fedratinib suggesting that the inhibition of JAK2 positive cells are not an off-target effect.

Dual Binding of SB 203580 to P38 MAP kinase

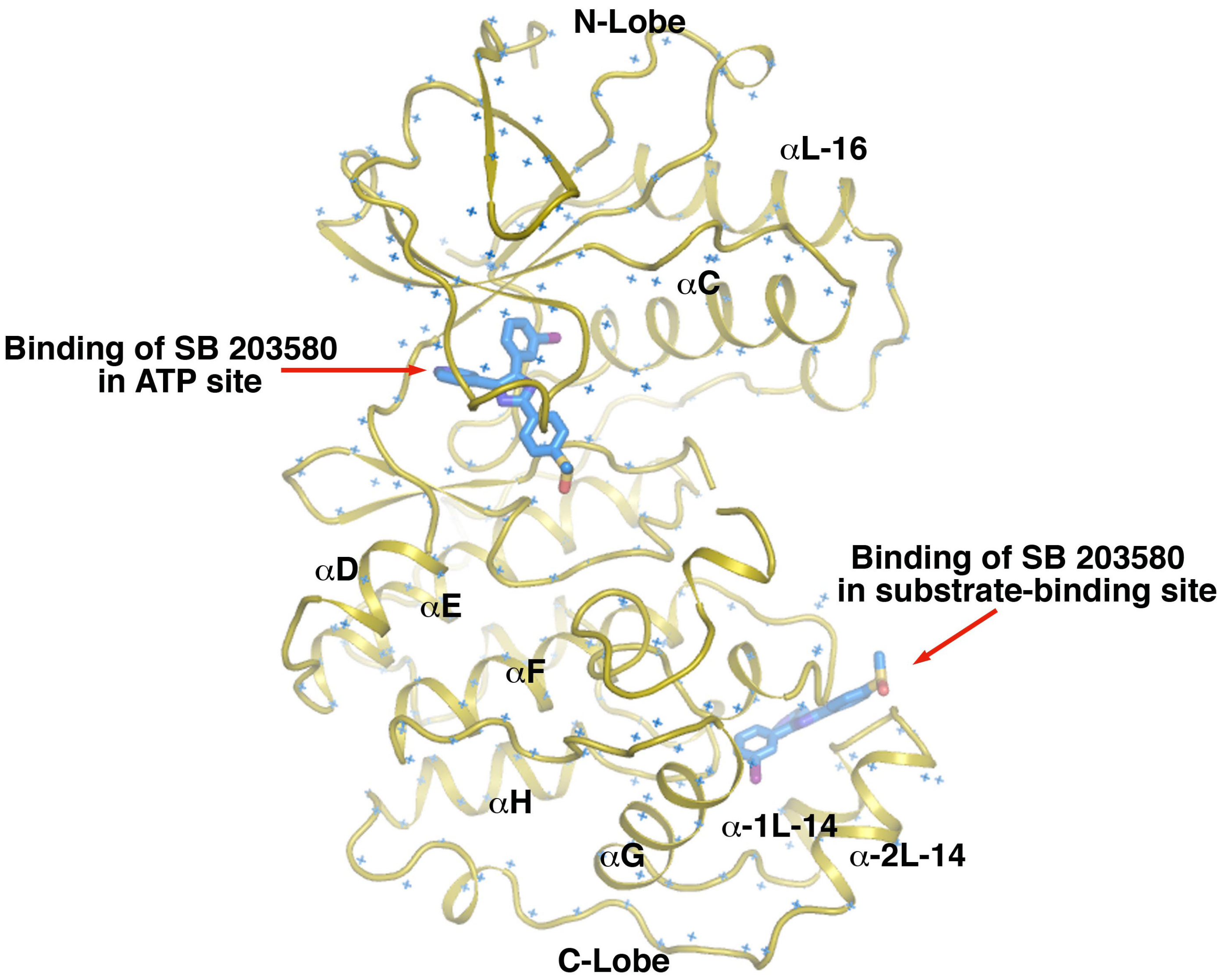

PDB ID: 1IAN

Tong et. al.. Nature Structure Biology. (1997). Volume 4, Number 4 , 311-316

Supplementary Figure 13: Dual binding of SB 203580 to P38 MAP kinase
A ribbon depiction of human P38 MAP kinase showing dual binding of SB203580.
Two inhibitor molecules are shown as blue sticks marked by red arrows indicating their respective binding sites.

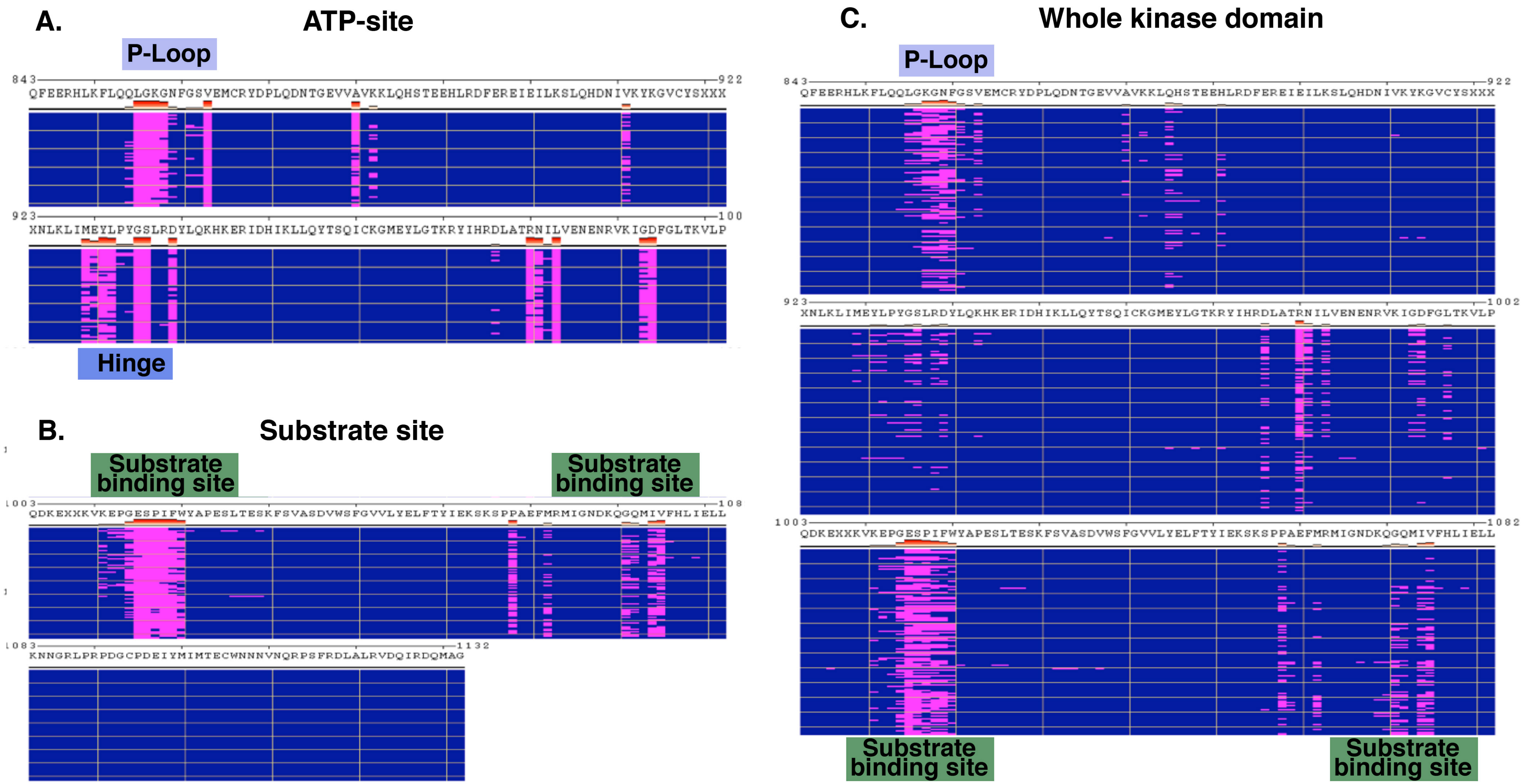

Supplementary Figure 14: Fedratinib binds to substarte binding pocket with higher affinity than the ATP site
Distribution of protein-ligand contacts in alternative poses of fedratinib, as predicted by multiple docking runs. Each row represents results for a distinct cluster of docking poses, as identified by AutoDock, with residues in contact with the ligand shown in magenta. The results for three different simulation boxes targeting the active (ATP binding) site, the substrate binding site and both sites together (using a large simulation box) are included in panels A, B and C, respectively. Note that the ligand shows preferential bonding to the substrate binding site whenusing the large simulation box that enables sampling of both pockets.
Polyview-MM (http://polyview.cchm.org) was used to generate the figures.

A

Fedratinib docking at ATP site

Distribution of the ligand docking models				
Rank / Order in the image (1 is the best)	Occupancy (number of simulation runs)	RMSD from the reference ligand structure, Å	Estimated Constant of Inhibition, Ki	Estimated Free Energy of Binding, kcal/mol
1	50	127.945	34.74 nM	-10.18
2	7	127.346	61.66 nM	-9.84
3	4	127.648	137.35 nM	-9.36
4	3	128.079	140.56 nM	-9.35
5	2	127.775	145.34 nM	-9.33
6	23	126.562	154.63 nM	-9.29
7	8	126.898	199.16 nM	-9.14
8	4	126.506	279.28 nM	-8.94
9	8	127.683	283.24 nM	-8.93
10	9	127.691	291.63 nM	-8.92
11	3	127.217	338.90 nM	-8.83
12	7	127.228	341.22 nM	-8.82
13	3	126.907	652.45 nM	-8.44
14	7	126.427	712.06 nM	-8.39
15	8	126.896	799.18 nM	-8.32
16	1	128.243	800.29 nM	-8.32
17	1	126.247	1.01 uM	-8.18
18	1	127.221	1.07 uM	-8.15
19	2	125.687	1.13 uM	-8.11
20	2	127.37	1.29 uM	-8.03
21	2	126.574	1.35 uM	-8.01
22	4	125.865	1.37 uM	-8
23	2	125.33	1.46 uM	-7.96
24	2	126.527	1.51 uM	-7.94
25	2	126.239	1.54 uM	-7.93
26	1	125.095	1.57 uM	-7.92
27	1	127.439	1.61 uM	-7.9
28	1	128.221	1.84 uM	-7.82
29	1	128.057	2.55 uM	-7.63

B

Fedratinib docking at Substrate site

Distribution of the ligand docking models				
Rank / Order in the image (1 is the best)	Occupancy (number of simulation runs)	RMSD from the reference ligand structure, Å	Estimated Constant of Inhibition, Ki	Estimated Free Energy of Binding, kcal/mol
1	7	116.348	129.36 nM	-9.4
2	3	112.936	258.99 nM	-8.99
3	1	111.605	350.89 nM	-8.81
4	1	113.808	416.06 nM	-8.7
5	3	116.416	563.67 nM	-8.53
6	12	110.895	565.47 nM	-8.52
7	9	112.712	668.63 nM	-8.42
8	1	113.594	994.36 nM	-8.19
9	7	112.441	1.44 uM	-7.97
10	6	113.802	1.55 uM	-7.93
11	2	109.632	1.58 uM	-7.92
12	4	112.913	1.72 uM	-7.86
13	2	115.367	2.06 uM	-7.76
14	2	110.298	2.11 uM	-7.74
15	1	111.42	2.20 uM	-7.72
16	6	113.839	2.80 uM	-7.57
17	4	113.151	2.90 uM	-7.55
18	1	113.737	2.95 uM	-7.55
19	4	112.359	3.12 uM	-7.51
20	12	113.009	3.26 uM	-7.48
21	2	113.243	3.29 uM	-7.48
22	3	111.852	3.32 uM	-7.47
23	1	111.442	3.75 uM	-7.4
24	1	113.635	4.07 uM	-7.35
25	3	112.012	4.52 uM	-7.29
26	1	110.913	5.04 uM	-7.23
27	1	110.896	5.16 uM	-7.21
28	2	111.571	5.22 uM	-7.21
29	1	113.528	5.36 uM	-7.19

C

Fedratinib docking ar whole kinase domain

Distribution of the ligand docking models				
Rank / Order in the image (1 is the best)	Occupancy (number of simulation runs)	RMSD from the reference ligand structure, Å	Estimated Constant of Inhibition, Ki	Estimated Free Energy of Binding, kcal/mol
1	2	120.502	321.47 nM	-8.86
2	1	118.88	343.69 nM	-8.82
3	3	116.104	370.03 nM	-8.77
4	1	128.661	378.37 nM	-8.76
5	1	126.866	612.88 nM	-8.48
6	5	116.102	647.04 nM	-8.44
7	1	114.656	744.10 nM	-8.36
8	15	116.373	764.99 nM	-8.34
9	1	126.635	1.15 uM	-8.1
10	1	118.297	1.30 uM	-8.03
11	2	126.604	1.38 uM	-7.99
12	1	114.088	1.39 uM	-7.99
13	5	117.348	1.55 uM	-7.93
14	1	117.296	1.59 uM	-7.91
15	1	115.999	1.59 uM	-7.91
16	2	119.033	1.63 uM	-7.9
17	1	119.298	1.66 uM	-7.89
18	1	117.657	1.73 uM	-7.86
19	1	127.301	1.96 uM	-7.79
20	1	117.32	2.18 uM	-7.72
21	1	123.754	2.28 uM	-7.7
22	4	113.167	2.28 uM	-7.7
23	1	125.953	2.33 uM	-7.69
24	1	119.601	2.43 uM	-7.66
25	3	115.861	2.49 uM	-7.64
26	1	110.136	2.82 uM	-7.57
27	1	108.733	2.82 uM	-7.57
28	2	117.906	2.95 uM	-7.54
29	1	125.632	3.12 uM	-7.51

Supplementary Table 2

Showing the binding free energy and constant of inhbition (Ki) in ATP pocket (A), substarte pocket (B) and in whole kinase domain that includes both pockets (C).

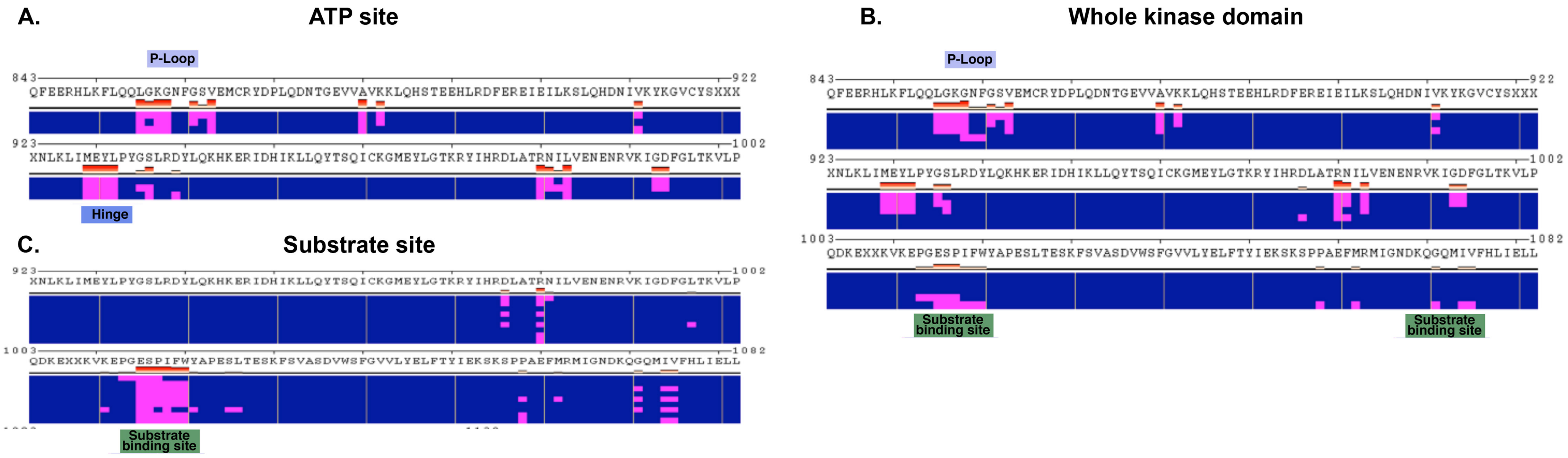

Supplementary Figure 15. Ruxolitinib preferentially binds to ATP site
Distribution of protein-ligand contacts in alternative poses of Ruxolitinib, as predicted by multiple docking runs. In analogy to supplementary figure 13, the results for three different simulation boxes targeting the active (ATP binding) site, the substrate binding site and both sites together (using a large simulation box) are shown in panels A, B and C, respectively.

Note that the ligand shows preferential bonding to the ATP binding site when using the large simulation box that enables sampling of both pockets.

A

Ruxolitinib docking at ATP-site

Distribution of the ligand docking models				
Rank / Order in the image (1 is the best)	Occupancy (number of simulation runs)	RMSD from the reference ligand structure, Å	Estimated Constant of Inhibition, Ki	Estimated Free Energy of Binding, kcal/mol
1	158	132.269	445.13 nM	-8.66
2	23	132.463	836.43 nM	-8.29
3	19	131.952	986.46 nM	-8.19

B

Ruxolitinib docking at substrate site

Distribution of the ligand docking models				
Rank / Order in the image (1 is the best)	Occupancy (number of simulation runs)	RMSD from the reference ligand structure, Å	Estimated Constant of Inhibition, Ki	Estimated Free Energy of Binding, kcal/mol
1	58	120.258	3.70 uM	-7.41
2	13	118.702	13.05 uM	-6.66
3	4	114.154	13.75 uM	-6.63
4	2	119.224	14.49 uM	-6.6
5	111	113.015	15.61 uM	-6.56
6	2	118.043	15.70 uM	-6.55
7	2	112.98	16.52 uM	-6.52
8	2	117.835	31.20 uM	-6.15
9	6	114.566	33.80 uM	-6.1

C

Ruxolitinib docking at whole kinase domain

Distribution of the ligand docking models				
Rank / Order in the image (1 is the best)	Occupancy (number of simulation runs)	RMSD from the reference ligand structure, Å	Estimated Constant of Inhibition, Ki	Estimated Free Energy of Binding, kcal/mol
1	140	132.24	504.37 nM	-8.59
2	35	132.445	937.24 nM	-8.22
3	23	131.903	1.12 uM	-8.12
4	1	120.249	4.29 uM	-7.32
5	1	112.91	15.02 uM	-6.58

Supplementary table 3

Showing the binding free energy and constant of inhbition (Ki) in ATP pocket (A), substarte pocket (B) and in whole kinase domain that includes both pockets (C).

A B

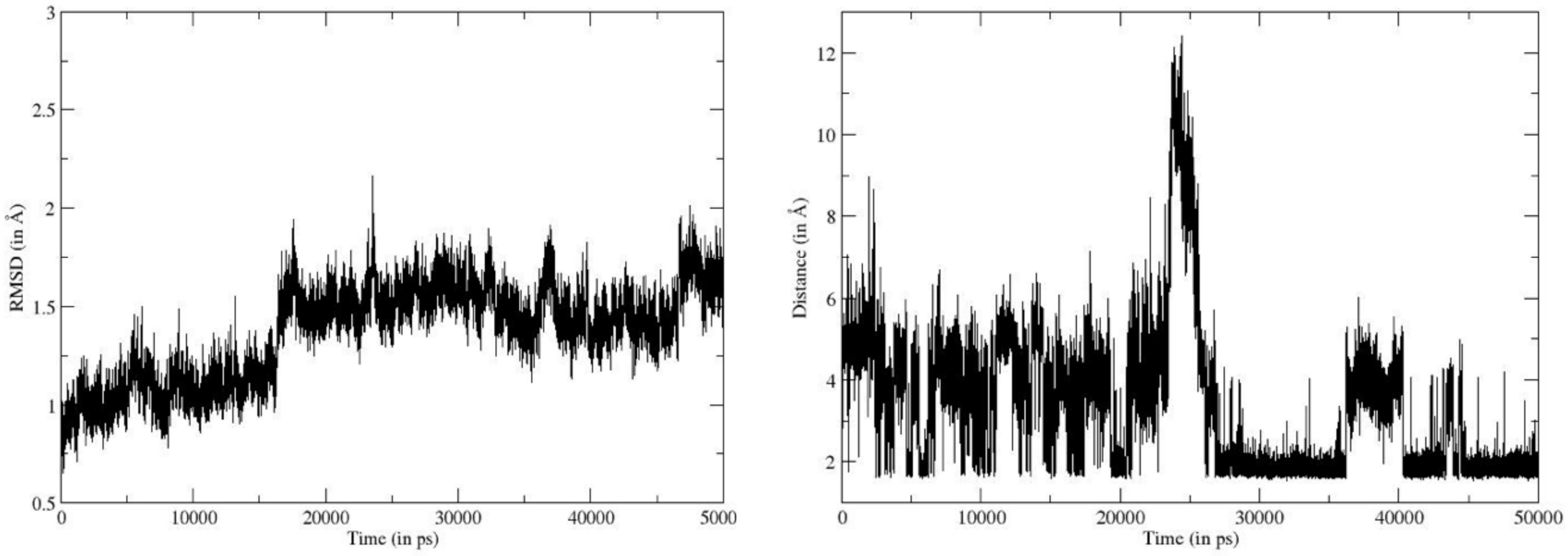

Supplementary figure 16. Molecular dynamics simulations provide further support for fedratinib binding to substrate binding pocket. The ligand remains in the substrate binding pocket in the course of 50ns MD simulations, as indicated by the formation of hydrogen bonds between the ligand and residues GLU1015, ASN859, and GLY858 that are observed in 39.5, 20.4 and 19.8% of 50,000 simulations snapshots, respectively.

A. Graph showing fluctuation of the protein-fedratinib complex during 50ns MD simulations, starting from the initial structure obtained by rigid protein docking, minimized and equilibrated, as described in the Methods section. Heavy atom root mean square deviation (RMSD) of the protein-ligand complex structure with respect to the initial structure is reported, with one structure saved in each ps. Note limited extent of fluctuations over the course of simulations, which indicates that the overall protein structure remains unchanged in the course of the simulations.

B. Fedratinib forms a hydrogen bond with Glu1015 in substrate binding pocket Graph showing the fluctuations of the distance between the hydrogen atom H37 of fedratinib and O atom of residue Glu 1015. Note the limited scope of these fluctuations over the course of the simulations, with the distance between the donor and acceptor atoms often approaching the theoretical length of the hydrogen bond of 1.8 Ang, especially in the second half of the trajectory when the formation of this hydrogen bond is observed most of the time.

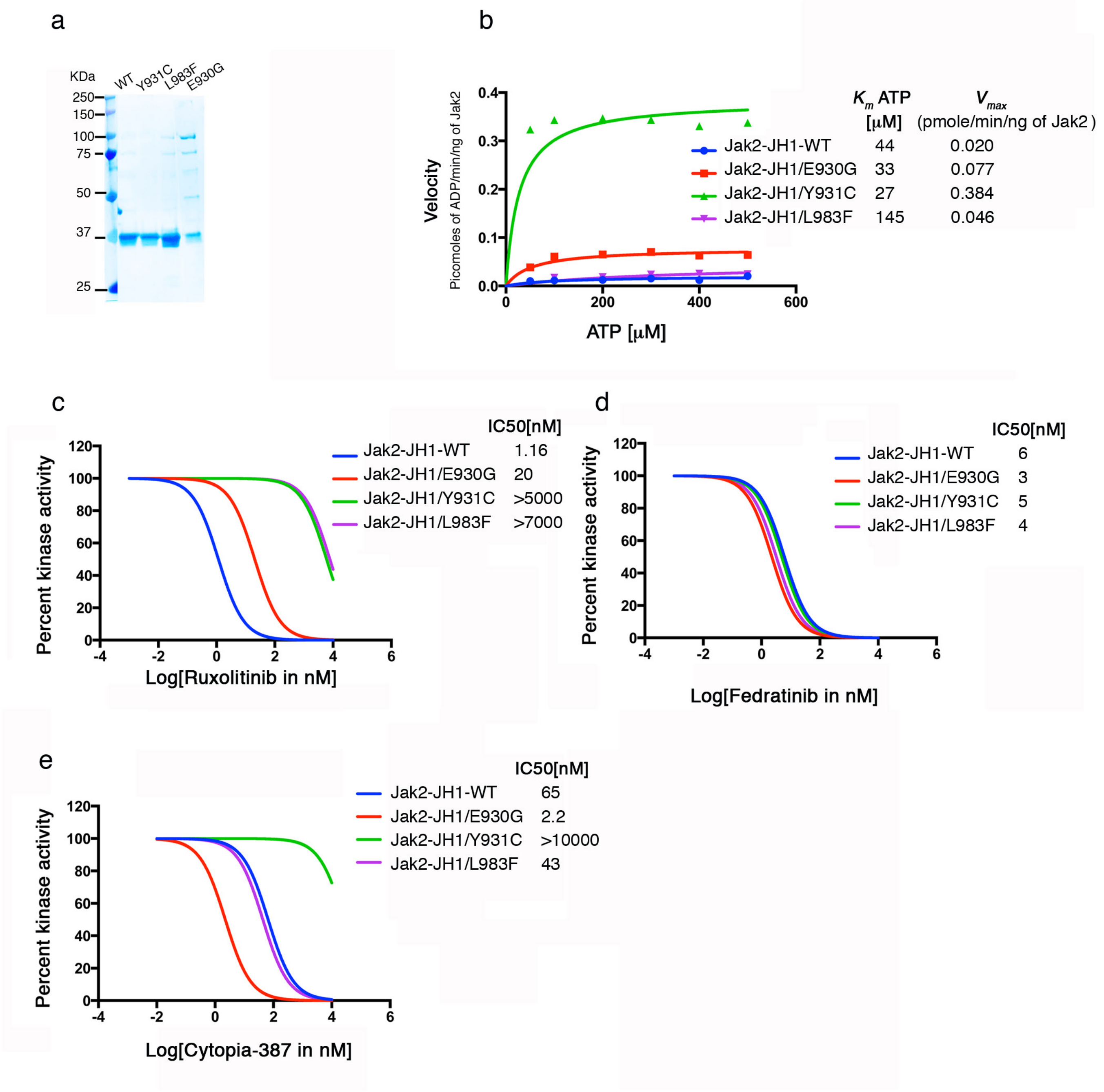

Supplementary Figure 17: Ruxolitinib resistant mutations from the hinge region of ATP binding site (Y931C and E930G) activate catalytic activity.and they are sensitive to Fedratinib inhibition.

a. A coomassie stained gel shpwing the purified kinase domain. Mutant E930G are expressed at very low level, and because of this reason purity of this mutant is ~80% while other variants were purified to >95% purity.

b. Steady state kinetics showing higher velocity for Y931C and E930G. Mutant Y931C is almost 20 fold more active than the wild type with ~ 2 fold decrease in Km for ATP.

c. Dose response analysis showing resistance to ruxolitinb by all three mutants but sensitive to fedratinib (d).

e. Dose response analysis against CYT-387 revealed that ATP site variants L983F and E930G are sensitive to inhibition but Y931C is fully resistant.

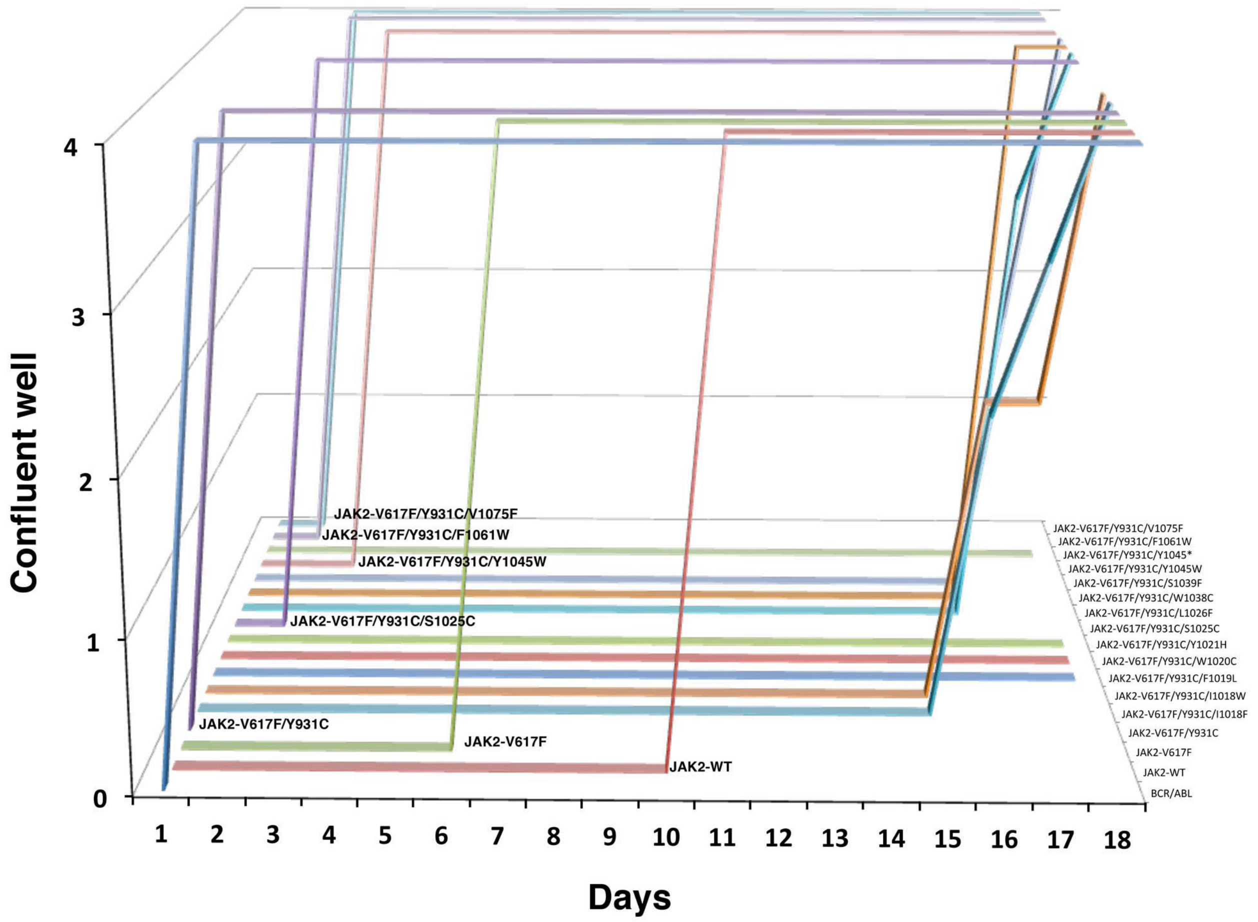

Supplementary Figure 18:

Mutations from the substrate binding site exhibit lower transformation potential than the parental Jak2-V617F/Y931C

Cell proliferation assay of BaF3 cells expressing BCR-ABL, Jak2-WT, Jak2-V617F and different variants of Jak2-V617F/Y931C. Cells were plated in quadruplicate in 96- well plates at a density of 5,000 cells per well in the absence of IL-3, and scored when the wells became confluent. BaF3 cellular transformation by oncogenic kinase for factor independent growth directly correlates with kinase activity. Please note that, mutations from the conserved IFW motif of athe activation loop expressed severly weakened kinase while others showing significantly reduced transformation viz kinase activation as comapred to Jak2-V617F/Y931C.

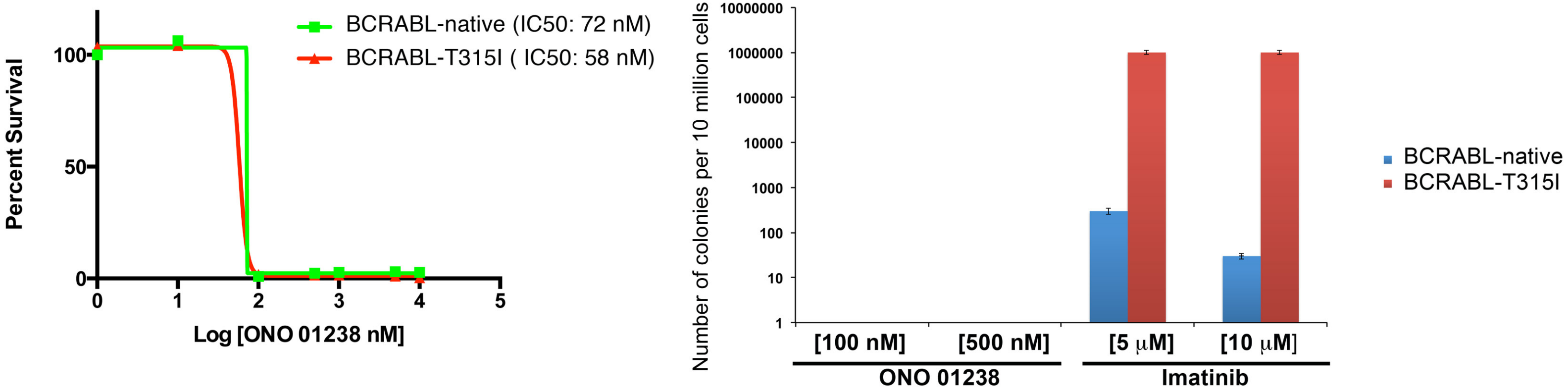

Supplementary Figure 19:
Lack of genetic resistance against substrate-site BCR/ABL inhibitor ONO 01238.

A. Dose response cell proliferation analysis of BCRABL and BCRABL-T315I showing dose dependent inhibition of cell proliferation. Of interest, BCRABL- T315I showing greater sensitivity than the native BCRABL, as T315I mutation will stabilize the active conformation that may favor for gerater affinity for substarte-site inhibitor.

B. Soft-agar colony formation assays showing the emergence of resistant clones against Imatinib at 5 and 10 μM of drug concentrations, representing 10 to 20 fold of cellular IC50, respectively..
While screening against ONO 01238 at 100 and 500 nM of drug concentrations representing 2 and 10 fold of cellular IC50 for the BCRABL, respectively, completely suppressed the emergence of drug resistance.
